# A sensorless, Big Data based approach for phenology and meteorological drought forecasting in vineyards

**DOI:** 10.1038/s41598-023-44019-4

**Published:** 2023-10-05

**Authors:** Ginevra Canavera, Eugenio Magnanini, Simone Lanzillotta, Claudio Malchiodi, Leonardo Cunial, Stefano Poni

**Affiliations:** 1 Dipartimento di Scienze delle Produzioni Vegetali Sostenibili, Via Emilia Parmense 84, 29122 Piacenza, Italy; 2 Latitudo Srl, Via Modonesi 12, 29122 Piacenza, Italy

**Keywords:** Physiology, Plant sciences, Climate sciences

## Abstract

A web-based app was developed and tested to provide predictions of phenological stages of budburst, flowering and veraison, as well as warnings for meteorological drought. Such predictions are especially urgent under a climate change scenario where earlier phenology and water scarcity are increasingly frequent. By utilizing a calibration data set provided by 25 vineyards observed in the Emilia Romagna Region for two years (2021–2022), the above stages were predicted as per the binary event classification paradigm and selection of the best fitting algorithm based on the comparison between several metrics. The seasonal vineyard water balance was calculated by subtracting daily bare or grassed soil evapotranspiration (ET_s_) and canopy transpiration (T_c_) from the initial water soil reservoir. The daily canopy water use was estimated through a multiple, non-linear (quadratic) regression model employing three independent variables defined as total direct light, vapor pressure deficit and total canopy light interception, whereas ET_S_ was entered as direct readings taken with a closed-type chamber system. Regardless of the phenological stage, the eXtreme Gradient Boosting (XGBoost) model minimized the prediction error, which was determined as the root mean square error (RMSE) and found to be 5.6, 2.3 and 8.3 days for budburst, flowering and veraison, respectively. The accuracy of the drought warnings, which were categorized as mild (yellow code) or severe (red code), was assessed by comparing them to in situ readings of leaf gas exchange and water status, which were found to be correct in 9 out of a total of 14 case studies. Regardless of the geolocation of a vineyard and starting from basic in situ or online weather data and elementary vineyard and soil characteristics, the tool can provide phenology forecasts and early warnings of meteorological drought with no need for fixed, bulky and expensive sensors to measure soil or plant water status.

## Introduction

Viticulture is facing new challenges worldwide due to global warming. Among them, the advancement of the growing season with anticipation of all main phenological stages, as well as the occurrence of more frequent and severe meteorological drought, is almost constant throughout the various vine regions^1–5^. Bud burst (e.g. commencement of vegetative growth), flowering and veraison—traditionally reached when berries start showing some pigmentation—are key phenological stages, and various studies have already examined their predictions through a modelling approach with a variable degree of accuracy^6–9^. Among them the dynamic crop model STICS^6^, mostly based on soil parameters, has been tested for three Portuguese grape varieties; however, correlation between observed and predicted dates for flowering was quite loose (R^2^ = 0.35). Temporal profiles of Normalized Different Vegetation Index (NDVI) have also been used at two sites in Portugal over a 8 year time span to infer flowering dates with a spread deviation of three days vs the ground based phenology^10^. Reliable prediction of budburst is needed more today than in the past, despite the number of frost days (i.e., the number of days in a year when air temperatures go below zero) generally diminishing due to global warming^11^; the impact of severe spring frost damage can still be dramatic or even more serious simply because bud burst is also greatly anticipated. Furthermore, in case the temperature drops below 0 °C, vegetation is at risk of almost reaching a stage of maximum hydration, resulting in a heightened sensitivity to frost^12–15^. Confusion can sometimes arise regarding the definition of “bud burst attainment” because it can mean the reach of different growth stages, ranging from a “swollen bud” (most common) to a “green tip” or "first unfolded leaf” stage. The estimates of the flowering date must deal with the typical asynchrony that patterns of flower opening display within the same cluster, vine and vineyard. In addition, flowering is bound to a quantitative estimate, since a value approaching the fraction of open flowers to total flowers is required to predict a date of occurrence. In red cultivars, the estimates of the onset of veraison, albeit still quantitative, are greatly facilitated thanks to color appearance. This is less helpful in white cultivars; however, regardless of the genotype, evaluating the onset of veraison on the basis of the start of pigmentation has been shown to lead to a rather delayed assessment. Older studies in this field have demonstrated that softness and surge in sugar accumulation are the two promptest signs of “veraison”, whereas color appearance and resumption of berry growth following the lag phase can be up to 5–6 days late^16,17^. A timely forecast of the onset of veraison is imperative, as from this date onward, predictions of final yield and full ripening usually become more accurate^17^. Another significant change in grapevine physiology with respect to veraison is that the water supply to the berry after veraison is primarily provided through the phloem, whereas prior to the veraison stage, xylem accounts for the incoming water budget of the berry^18^. Furthermore, it has been found that the grapevine berry after veraison acquires more tolerance to drought as the hydraulic communication between the shoot and the cluster is partially impaired^19^.

Global warming is increasingly urging the need to provide reliable alarms to growers in terms of the early occurrence of significant meteorological drought, including a combination of moderate-to-low soil moisture and high evaporative demand in the summer months. In this case, the demand is not mainly referred to dry areas where, due to almost a total lack of summer precipitation, the irrigation practice is ordinary and quite consolidated. Instead, it refers to temperate and even semi-continental growing areas, which have increasingly been facing issues of summer drought and show a decreasing return time, especially in France and Italy^1,20,21^. In these regions, where chances of having erratic abundant precipitation are high in summer, the more urgent need is not the amount and modality of water supply through irrigation; rather, it is the prediction of if and when significant water stress will occur. In this matter, increasing recourse to decision support systems (DSSs) aided either by new interconnected sensors pertaining to the Internet of Things^22^ or Big Data analytics^23^ represents a new frontier. However, it should be agreed that efforts made insofar to implement DSSs in viticulture and other crops have been primarily focused on assisting decisions related to pest and disease management with an overall good rate of success^24–27^. Conversely, when the prediction is focused on abiotic stresses, especially water stress involving all related aspects of irrigation management, contributions are lower in number and more uncertain^25,28^. A survey conducted in Canada on multiple crops spread across 12 irrigation districts in Alberta depicted that out of 199 participating farmers, only about 1% showed convinced DSS adoption for improved use of irrigation, whereas the large majority still relied upon visual and direct assessment such as appearance and feel of soil moisture to guide their decision-making^29^. In a vineyard, this might correspond to the evaluating degree of leaf inclination or the curving of the growing tip.

Reluctance toward DSS adoption is indeed partially due to the farmers’ resistance to give up their decision-making processes. This presents an even greater hurdle in viticulture, where, especially in the Old World, the tradition of millenary crops exceptionally bound to tradition and family-inherited rules is difficult to be scratched^30^. However, a significantly possible reason accounting for farmers’ limited adoption of DSSs is that most of the time, the farmers are under the pressure of a technology push rather than the demand of end users who would like to solve a specific problem or improve a practice^31^.

Regarding viticulture, many specific reasons can be presented for an overall modest adoption of DSSs for stress detection and irrigation management. Oftentimes, the problem resides in the choice of physiological parameters (i.e., stomatal conductance), which are too fine and sophisticated to be implemented into a DSS^32^. This latter DSS was improved when a threshold of the free transpirable soil water was taken into account (0.4), beyond which no reduction in vine transpiration was considered^33^. In other cases, DSSs have proved successful at simulating the soil water balances of various soil types within an area of interest^34^; however, they lack accuracy in terms of actual water use by the vineyard ecosystem. A successful large-scale DSS application for on-farm irrigation is IRRINET^35^, which is mostly tailored to extensive field crops.

A combination of desirable features must be fulfilled to decidedly increase the adoption of DSSs and the management of water relations in vineyards. These features include service-based, reasonably low-cost and acceptable accuracy on the warnings; warning of pre-stress threshold to be released with text messages sent to a mobile phone; clear guidelines to be provided for vineyard scouting eventually required to confirm or dismiss the alarm; no direct data manipulation or processing required by the end users in case they are limited to simple inputs aimed at improving output accuracy (i.e., entering the actual date of an observed phenological stage); and minimum or no presence of bulky and costly sensors for assessment of soil or plant water status variables to be used or processed to produce the stress warnings. The latter feature is crucial to growers as they can hardly bear having fixed obstacles during routine vineyard operations, which implies interaction with the row hindrance (e.g., mechanical shoot trimming, under-the-row tillage and mechanical harvesting). Although the robustness and stability of vine or leaf permanent sensors deputed to continuously track the plant water status have improved^36–38^ their presence is still perceived as an additional bother, and damages due to incautious interventions are frequent. On the other hand, water-stressed alarms sent on the basis of limiting soil moisture or soil water potential thresholds reached by permanent sensors placed at various soil depths are perceived as too bulky and invasive while causing doubt that a few measuring points in an area (for instance a 1 ha vineyard), which is often characterized by high intra-vineyard spatial variability based on soil heterogeneity, might not be representative of the true vineyard water status^39,40^.

Thus, if the new frontier is using the vine (canopy) as a main sensor to achieve the most simplified and accepted DSS, then the role of Big Data analytics becomes important^41–43^. Regardless of its size, any vineyard farm accumulates data from various resources including ground sensors (with weather stations being the most common), statistical yearbooks, real-time web data from private companies through online web services, reports from grapes delivery to wineries, a crowdsourcing-based technique from mobile phones and feeds from social media^23^. Techniques and tools required for Big Data analytics include machine learning, cloud-based platforms, image processing, modeling and simulation, statistical analyses and Normalized Difference Vegetation Index (NDVI) vegetation indices^44–46^.

Newlands^46^ has recently published a comprehensive review covering many use-cases of co-application of Artificial Intelligence (AI) and Big Data in viticulture and enology. Use cases include bunch detection, vineyard management, disease and pest control, biotic and abiotics stresses, phenology and yield prediction, wine aroma, traceability, authenticity and protection. Focusing on the phenology and water stress assessment also taken into account in our study, the most relevant were a DSS support system coupling fuzzy logic and probabilistic graphical approaches to predict technological ripening in grapevine berries^47^ and a spatial model aimed at describing spatial variability of grapevine phenology at the within-field scale through a single measurement performed in the field (reference site) and a combination of site-specific coefficients calculated through historical information^48^. Model calibration carried out on two cultivars grown in a small vineyard observed for a total of six seasons led to a RMSE of about three days for bud burst, flowering and veraison estimates; however, the same authors conclude that if the variability of phenology is low, the traditional method of sampling could lead to better results.

Turning into use cases dealing with abiotic stresses^49^, the two most relevant applications refer to modeling of water stress in cv Sirah based on hyperspectral imaging and machine learning^50^ and to assessing the feasibility of using Sentinel-2 imagery to quantify the impact of heatwaves on irrigated vineyards^51^. New decision-support tools have also been developed that use Big Data and AI technology. A good example is Vitiapp™ a pre-commercial web-based application for supporting decisions about vineyard management^52^. It includes environmental data (weather, soil) to describe conditions influencing grape yield and fruit composition, cloud computing to integrate multiple data streams from a diversity of vineyard sensors and weather forecast data. It provides vineyard patch-specific awareness of weather-based risks for each selected management issue: botrytis/powdery/downy disease, and frost/chilling/heat accumulation, wind, rainfall, soil moisture and/or spraying conditions.

The main objective of this study is the description, calibration and validation of a new online Big Data based tool aimed at providing predictions for phenology and meteorological drought in vineyards, which, in a sensor less perspective, is exclusively based on the elementary inputs of the vineyard ecosystem and the weather climatic data.

## Material and methods

### The vineyards’ characteristics and description

For this study, a total of 25 vineyards were monitored in 2021 and 2022 (Table 1), and 22 of them (V1–22) were used for the calibration of the modelled budbreak, flowering and veraison dates, whereas the remaining three (V23–25) were surveyed for validation of the drought warnings. The vineyard sample, including traditional, organic and biodynamic management, allowed for the following variability expressed as possible types or min-to-max intervals: nine cultivars and three rootstocks aged 9–33 years; cane and spur pruning of size 0.23–2.7 ha; four row orientations (EW, NS, NE–SW, NW–SE); six exposures (S, N, SE, W, NW, SW); between-row vine spacing of 2.0–3.4 m; in-the-row vine spacing of 0.75–1.50 m; vine density of 2500–5000/ha; canopy height and thickness at full development and upon shoot trimming at 0.9–1.4 m and 0.30–0.50 m, respectively; soil texture (clay-loam, silty-clay loam and clay); interrow soil management (tillage, spontaneous grass, alternate tillage and spontaneous grass, winter cover crop terminated in spring by rolling); under-the-row soil management (herbicides, tillage, mowed spontaneous grass); irrigation present in 3 out of 25 vineyards; and altitude at 27–430 m a.s.l. (Table 1). Of the nine cultivars included in the calibration/validation data set, two of them (Pinot noir and Ortrugo) usually show an early budburst, whereas the remaining’s have a quite variable behavior.

**Table 1:** Main characteristics of all vineyards monitored for calibration and validation of the Big Data outputs.

Vineyard code	Farming system	Scion/roostock	Year of planting	Training system	Vineyard surface (ha)	Row orientation	Vineyard exposure	Between-row spacing (m)	In the row spacing (m)	Vine density/ha	Maximum canopy height (m)	Maximum canopy width (m)	Soil texture	Between-row management	Under the row management	Irrigation	Min and max altitude (m a.s.l.)
V1	Organic	Ortrugo/K5BB	2010	Guyot	1	NW–SE	SE	2.25	0.9	4000	1.2	0.4	Clay loam	SG	SG	No	249–267
V2	Organic	Malvasia di C.A./K5BB	2010	Guyot	0.8	NW–SE	SE	2.25	0.9	4000	1.2	0.4	Clay loam	SG	SG	No	265–248
V3	Organic	Pinot Nero/SO4	1989	Cordon	0.4	NW–SE	SE	2.25	0.9	4000	1	0.4	Clay loam	SG	SG	No	245–229
V4	Organic	Barbera/K5BB	2005	Cordon	0.7	N–S	_	2	1	5000	1.25	0.40	Silty-clay-loam	SG	T	No	260–254
V5	Organic	Croatina/K5BB	2006	Guyot	0.3	N–S	_	2	1	5000	1.25	0.40	Silty-clay-loam	SG	T	No	254–249
V6	Organic	Sauvignon blanc/1103P	2007	Guyot	0.23	E–W	E	2	1	5000	1.25	0.40	Silty-clay-loam	SG	T	No	260–246
V7	Organic	Malvasia di C.A/K5BB	2007	Guyot	0.31	E–W	E	2	1	5000	1.25	0.40	Silty-clay-loam	SG	T	No	257–252
V8	Conventional	Ortrugo/SO4	2007	Guyot	2.5	NW–SE	N	2.5	1.2	4800	1.30	0.40	Silty-clay-loam	Alternated SG /T	T	No	430–408
V9	Conventional	Malvasia di C.A./SO4	2002	Guyot	1.5	NW–SE	SE	2.5	1.2	4800	1.15	0.30	Silty-clay-loam	Alternated SG /T	T	No	410–384
V10	Conventional	Croatina/K5BB	2013	Guyot	2	NW–SE	SE	2.5	1.2	4800	1.25	0.40	Silty-clay-loam	Alternated SG /T	T	No	402–392
V11	Conventional	Barbera/K5BB	2013	Guyot	2	NW–SE	SE	2.5	1.2	4800	1.25	0.40	Silty-clay-loam	Alternated SG /T	T	No	405–396
V12	Bio-dynamic	Sangiovese/1103P	2003	Cordon	2	N–S	S	3	1.2	4000	1.20	0.30	Silty-clay-loam	T	T	No	109–82
V13	Bio-dynamic	Sangiovese/SO4	2002	Guyot	2.2	NE–SW	SW	3.2	1.2	3750	1.10	0.45	Silty-clay-loam	T	T	No	115–86
V14	Organic	Sangiovese/1103P	2004	Guyot	2.70	NW–SE	NW	3.4	1.2	3529	1.10	0.30	Silty-clay-loam	T	T	No	104–89
V15	Organic	Trebbiano/1103P	2008	Guyot	2.14	E–W	W	3.4	1.5	4412	1.10	0.30	Silty-clay-loam	SG	T	No	137–106
V16	Bio-dynamic	Trebbiano/K5BB	2011	Guyot	1.56	NW–SE	E	3	1.25	4167	1.10	0.45	Clay loam	SG	H	Yes	96–69
V17	Bio-dynamic	Sangiovese/SO4	2002	Guyot	2	NW–SE	E	3	1.1	3667	1.10	0.40	Clay loam	SG	H	Yes	84–65
V18	Bio-dynamic	Trebbiano/K5BB	2010	Guyot	0.4	NE–SW	_	3.3	1.5	4545	1.10	0.50	Silty clay loam	SG	H	Yes	27–27
V19	Organic	Barbera/420A	2006	Guyot	0.8	NW–SE	SE	2	0.75	3750	0.9	0.4	Clay loam	SG	T	No	269–237
V20	Organic	Malvasia di C.A./SO4	1990	Guyot	0.989	NW–SE	SE	3	0.75	2500	1.3	0.4	Clay loam	SG	T	No	251–242
V21	Organic	Barbera/SO4	2006	Guyot	1.6	N–S	S	2	0.75	3750	1	0.4	Clay loam	SG	T	No	250–228
V22	Organic	Malvasia di C.A./K5BB	2006	Guyot	0.495	N–S	S	2	0.75	3750	1	0.4	Clay loam	SG	T	No	251–235
V23	Organic	Malvasia di C.A./SO4	2016	Guyot	0.6	NW–SE	_	2.3	1	4348	1.4	0.35	Clay-loam	SG	M	No	154–151
V24	Organic	Pinot nero/SO_4_	2008	Guyot	0.58	NW–SE	NW	2.2	1	4545	1	0.25	Clay	SG	M	No	287–262
V25	Organic	Barbera/420A	2001	Guyot	0.4	NW–SE	_	2.4	1	4167	1.2	0.3	Clay-loam	SG	M	No	220–217

Vineyard locations ranged from 44° to 45° in latitude and 9° to 11° in longitude. Last three vineyards (V23-V25) were used for validation of the water stress alarms. *SG* spontaneous grass, *T* tillage, *H* herbicides, *M* grass mowed.

### Big Data analysis

**Figure 1:**
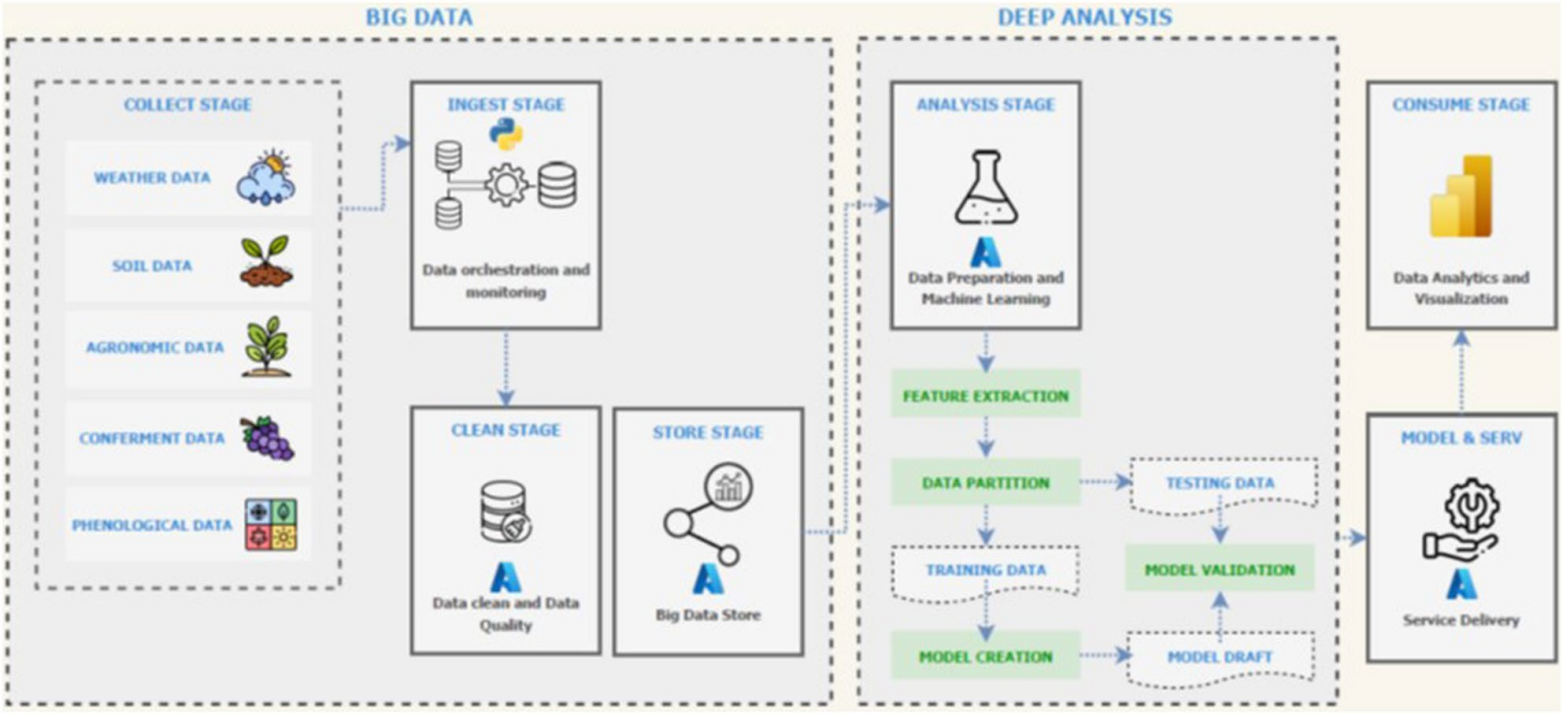
Data flow and processing used in the Big Data analytics leading to the Big VINE app.

The data flow rigorously followed a Big Data analysis framework (Fig. 1), which involved an initial phase of data collection from various sources followed by migration and storage, after a proper cleaning phase. The data were then used for model creation and final visualization of the outputs.

The data collected from the various vineyard locations and farmers (Table 1) included five distinct data families, i.e., daily weather data (2012–2022), one-time vineyard characteristics data (e. g. vine spacing, soil texture, etc.), grape delivery data (2012–2022), and phenological data (2021 and 2022). The data collection (except for the weather data) was carried out by providing the project partners with a list of Excel files containing well-defined table structures to be compiled. Once the collection was complete, the flow moved onto the ingest stage, which consists of the migration of data to a centralized cloud environment. The storage and clean stages thus take place to assess the stored and organized data, allowing the viewing of various data sources and fast checking of preliminary correlations found between them. In this study, the Microsoft Azure platform, particularly the Azure SQL Database, for the creation of the cloud Datawarehouse (DWH) was used. This is a system designed to enable and support business intelligence activities, especially analytics. As an architecture, the Data Staging Area was chosen^53,54^ for the data collection phase, with a data cleaning and validation process following it, guaranteeing the integrity and completeness of the data. Hence, a staging area was defined on the DWH to apply all the various operations that define the clean stage, as well as all the extraction, transformation and loading operations.

Relevant actions applied to the Data Cleaning phase were: (i) Data Collection—gathering data from various sources which might be representative of the addressed issue; (ii) Data Exploration—it encompasses data structure and quality, data visualizations, graphs, and descriptive statistics with the goal of identifying missing values, outliers, potential issues in data distributions and patterns or trends; (iii) Handling Missing Data—in cases where raw data contains missing or null values, actions were removing rows or columns with missing data or imputing missing values using statistical methods (such as mean or median imputation); (iv) Handling Outliers—in cases where raw data contains outliers (values significantly different from the majority of data) that could negatively impact the ability of a Machine Learning model to make accurate predictions, it could be decided to remove, transform, or label them for further analysis. In our case, no significant outliers were detected, and the dataset was kept unchanged; (v) Normalization and Standardization—to ensure that variables have the same scale, data standardization was applied to prevent chosen algorithms from being affected by differences in scale between variables; (vi) Feature Selection—this process aims to identify and retain only the most informative features, reducing noise in the data. It implies a dimensionality reduction process through which extracting the best features from large data sets is possible by selecting and combining variables, thus effectively reducing the overfitting risk in a machine learning model. Initial cut-off used in our study was to maintain those having a Pearson correlation coefficient (r) > 0.5 or − 0.5 when regressed over occurrence of budburst, flowering and veraison. However, this would have led to an excessive reduction in the number of the training variables and, thus, cut-off was taken at r > 0.3 or − 0.3; Data Splitting—finally, the cleaned data is split into the train set, used for the creation and training of the various models, and the test set, used for model validation. The two subsets were created by randomly taking the records from the dataset under a train/test ratio of 80:20. In supervised Machine Learning, reserving 80% for training and 20% for testing is the most frequent choice according to the Pareto principle (also called the 80–20 rule), which states that 80% of the effect is driven by 20% of causes (and vice versa)^56^. Hereafter, potentially suitable machine learning algorithms (Random Forest, Support Vector Machine, K-Nearest Neighbours and XGBoost)^55^ for the prediction problem addressed were considered. The choice of these machine learning models was driven by their characteristics, including their resistance to overfitting, their ability to handle large datasets, and their computational efficiency. Once trained on the training set, they were evaluated in terms of performance with the test set using the AUC-ROC, Accuracy, Recall, Precision F1-Score metrics and then selecting the best ones to produce the phenological predictions. Model accuracy was given as Root Mean Square Error (RMSE) exploiting the advantage of predictions being expressed in days. Once an environment was defined to store, orchestrate and monitor data and the analysis and modeling phases had ended, the real use of data through business intelligence platforms became feasible. Within the Microsoft framework, the Power BI interactive platform was chosen to provide cloud-based capabilities and interactive visualizations, with an interface simple enough for end users to create reports and dashboards.

### Phenology assessment and modelling performance and accuracy

The assessment of the occurrence of budburst, flowering and veraison was performed in 2021 and 2022 on a sample of ten randomly chosen vines per vineyard (V1-V22 in Table 1). According to Baggiolini’s classification, as reported in Coombe^56^, attainment and crossing of stage B (defined as swollen bud) were taken as the criterion to assess that budburst had been reached. On each vine, the observed buds were those borne on the fruiting cane; in the case of a spurred cordon system, they were classified as count buds. Each year, the visual estimates began on V1–22 when all the buds were dormant, and a two-day assessment interval was kept for maximum accuracy of the estimate. Budburst was declared to have been reached when at least 50% of the observed buds in at least 50% of the tagged vines had reached or crossed stage B.

Assessment of flowering (stage I) date inherently required a quantitative approach, which was performed by a trained crew. On each test vine, two shoots were tagged, i.e., one basal and one apical on the cane pruned systems or one apical of each of the two spurs located in the basal and apical portion of the cordon. The ratio of open flowers to total flowers was visually assessed at two-day intervals. Flowering was declared to have been reached when at least 50% of the observed inflorescences on each vine had crossed 50% of open flowers.

Assessment of veraison (stage L) was performed on the clusters assessed for flowering attainment. The assessment consisted of finger-touch evaluation on three berries per cluster that were randomly chosen from the top, mid and bottom portions of the clusters and classified as either hard or soft. The survey was conducted at two-day intervals, and veraison was declared to have been reached when at least 50% of the observed berries on at least 50% of the tagged vines were classified as “soft”.

Prediction of the occurrence of budburst, flowering and veraison stages followed the binary event classification paradigm^57^, where each date could have only two possible outcomes: “1” if a phenological event was recorded on that date and “0” otherwise. It was also decided to create a distinct model for each phenological phase to apply the technique of oversampling (i.e., an increase of cases of positive class) and under-sampling (i.e., a decrease of cases of negative class)^23^. For the model selection, a benchmark was created to compare some of the most used frameworks for this type of problem. At the end of the evaluation performed using the precision, recall, accuracy, AUC-ROC and F1-score metrics^23^, the best-performing model for each phenological stage was selected. However, to better understand the meaning of the metrics, the concept of the Confusion Matrix must be defined^23,58^. In a binary classification system, when the model is asked to distinguish between two classes (e.g., positive or negative), four possible outcomes are identified: true positives (TP), an outcome where the model correctly predicts the positive class; true negatives (TN), an outcome where the model correctly predicts the negative class; false positives (FP), an outcome where the model incorrectly predicts the positive class; and false negatives (FN), an outcome where the model incorrectly predicts the negative class. Precision refers to the number of true positives divided by the total number of positive predictions (i.e., the number of true positives plus the number of false positives), thereby providing an estimate of model accuracy to identify a given class. Recall is calculated as the ratio between the number of positive samples correctly classified as positive to the total number of positive samples. Hence, it measures the model’s ability to detect positive samples. Accuracy is defined as the fraction of correct predictions out of all predictions made, and as such, it provides an estimate of the overall model performance. Finally, the F1-score integrates the combined result of precision and recall, since it represents their harmonic mean (F1-score = (TP)/(TP + 1/2(FP + FN)), where the false negatives and false positives are terms of the divider; therefore the lower they are, the higher the value of the F1 Score is; the higher the F1 Score value, the better the model performs. The metric used for model selection included the root mean square error (RMSE), defined as a weighted measure of model accuracy given on the same scale as the prediction target (i.e., days in the present study). RMSE is the average error that the model’s predictions possess in comparison with the observed values.

To validate the phenological models, the leave-one-out cross-validation procedure^59^ was applied during the training phase. This implies having n observations in the dataset, wherein all observations except one are taken: the model is trained iteratively on all the n-1 observations and validated against the only excluded observation. This process allows to train the model to limit the problem of over fitting while increasing the generalization capacity of the model itself. At the end of the training phase, all the benchmark models were subjected to the test phase using the test set, which consisted in the randomly selected units V6, V10, V19. The prediction of the phenological event was based on the future time horizon provided by the weather data source (one week) and on a pre-established likely time window for each phenological event defined as follows: from Day of Year (DOY) 65 to 100 for bud burst; from DOY 140 to 175 for flowering; and from DOY 190 to 230 for veraison. Once these time windows were entered into the model, the designated model was queried for the extraction of the prediction. Due to the binary classification environment, an a priori threshold was set to allow evaluation if a prediction was sufficiently reliable to be considered as a possible date for the event. If several possible dates were identified within the pre-established time windows, the average of the possible DOYs was calculated. Thermal time elapsing between two given phenological stages (e.g. bud burst to flowering) was expressed using cumulated growing degree day (GDD) which, in its most common expression, will use a base 10 °C temperature threshold^60^.

As per drought warnings visualization, Big-VINE uses a stoplight fashion, wherein green corresponds to no stress, as indicated by soil retaining > 60% of soil available water (SAW); yellow warns for mild stress, when 60% < SAW > 40%; and red accounts for severe stress (SAW < 40%).

### Calculation of under canopy shaded area and total canopy light interception

A shared definition of a crop coefficient (k_c_) is the ratio of actual crop evapotranspiration to reference crop evapotranspiration^61^. Grapevine water use and the k_c_ are linear functions of the shaded area which is measured beneath the canopy^62^. The latter was calculated in this study by feeding equations and functions which are listed in Table S1 into a custom-built Mathcad computer program (PTC, Boston, US). Moreover, a few elementary vineyard features, such as geographical coordinates, main slope, exposure, row orientation, maximum canopy thickness, maximum canopy height and distance between rows (Table 1), were also assessed. As supported by Ayars et al., Poni et al. and Iandolino et al.^63–65^, the ratio (%) between under-canopy shaded area and total area (depending upon between-row spacing) is considered a reliable estimate of total canopy light interception (TCLI). Therefore, the software can calculate the intercepted radiation parameters of any vineyard, including the variability due to slope and aspect.

**Figure 2:**
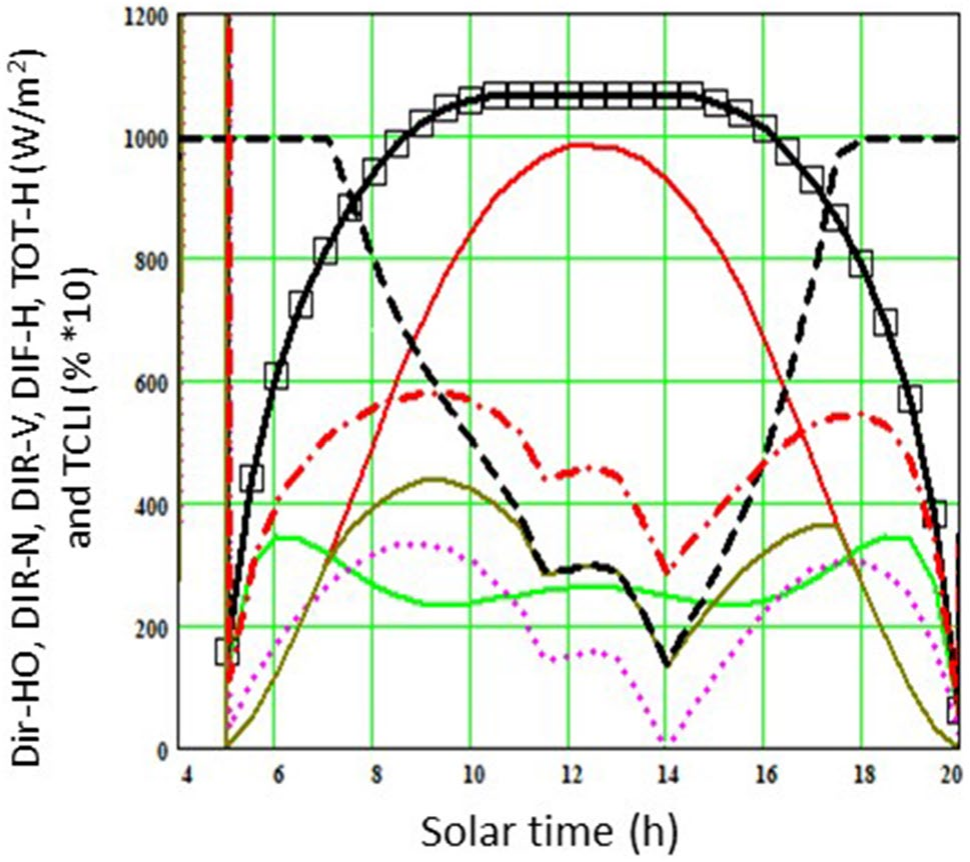
Simulation of diurnal trends of total direct light on a horizontal surface (DIR-HO, red line), total direct light normal to a surface (DIR-N, squares), total direct light on a vertical surface (DIR-V, violet broken line), total diffuse light on a vertical surface (DIF-H, green line), total direct and diffuse light on a vertical surface (TOT-H, dark green) on a 35° NE-SW oriented row calculated on 20 July (DOY 201) by Mathcad PLUS 6·0 software (Mathsoft Inc., Cambridge, MA, USA). Parameters used in the multiple non-linear regression model were total canopy light interception (TCLI) and DIR-V.

To provide an example of the type of graphical outputs that the software delivers, Fig. 2 depicts diurnal patterns of direct and diffuse solar radiation hitting a vertical shoot positioned trained vineyard located in Piacenza (45.1°N, 9.6°E, Italy). The simulation refers to 20 July 2022 (DOY 201).

### Calculation of modelled canopy water use

Under the assumption that TCLI is not necessarily a good estimator of daily canopy transpiration (for instance, early morning or late afternoon high TCLI values due to low solar angles do not usually correspond to high canopy transpiration since incoming radiation and vapor pressure deficit are still limiting), a multiple linear and nonlinear (quadratic) regression approach was used to infer the canopy transpiration (T_C_ as y) from the modelled TCLI (%, independent variable x_1_); amount of incoming direct light (µmol m^−2^ s^−1^, independent variable x_2_); and measured air vapor pressure deficit (VPD) as kPa, independent variable x_3_. Parameterization of the multiple linear and non-linear regression models was performed over a period of 16 days between 16 June and 10 August, when the gas exchange system was also in place and operating on a 24-h basis.

### Measurements of soil and cover crop seasonal water use

As the contribution to the vineyard ecosystem water budget from any resident or sown cover crop was reported to be significant^66,67^, this fraction was assessed in one (V25) of the three vineyard sites used for the drought warnings validation. In both test years (2021 and 2022), these vineyards shared the same floor management, implying a temporary winter grass sown in the fall between rows, which was terminated early spring through rolling, while repeated tillage under the trellis was used to maintain a mostly weed-free strip. In the 2021 season, sowing dates were 8 October 2020 for V23 and V25 and 9 October 2020 for V24, and grass termination was performed on 18 May 2021 and 19 May 2021. In 2022, the sowing dates were 14 October 2021 for V23 and V25 and 15 October 2021 for V24. Grass termination occurred on 18 May 2021 (V23 and V24) and 19 May 2022 (V25), respectively. In all cases, interrow grass was terminated using the ECO-ROLL mulch roller (Clemens, Wittlich, Germany). The ECO-ROLL compresses the vegetation, whereupon the spades mounted on the roller kink the stalks and fold them over. The result is a thick mulch carpet that progressively dries out, leaving little room for the resumption of native vegetation. In each season and vineyard, the same grass mixture was used at an 80 kg/ha sowing rate to correspond to the commercial product Humusfert (Padana Sementi, Padova, Italy), with 45% of *Veccia sativa* var Alexandros, 15% *Veccia villosa* var Haymaker Plus, and 40% *Avena nera* sativa var Gniady. In 2022, cover crop transpiration and soil evaporation rates were determined in the V25 Barbera vineyard with the portable close-type chamber system described and validated in Capri et al.^68^. The readings were taken four dates prior to and after grass termination (seven days before and 1, 14 and 30 days after) on ten different ground spots representative of tilled and rolled areas. To achieve a more reliable diurnal trend, each date readings were conducted at 10 and 12 AM, as well as at 1, 3 and 5 PM. The data were given as mm/day, and the derived pattern was extended to the other vineyards and seasons.

### Calibration of canopy light interception and transpiration

To provide proper calibration of the model-derived TCLI and T_C_ values, field measurements were conducted in a small experimental vineyard at Residenza Gasparini, a facility of the Università Cattolica del Sacro Cuore located in Piacenza (45.1°N, 9.6°E, Italy). The vineyard features four 60 m long, 35° NE–SW oriented Cabernet Sauvignon (*Vitis vinifera* L.) rows grafted onto SO4 rootstock. The spacing is 2.5 m × 1 m (between rows and in the row, respectively), with a resulting density of 4000 vines/ha. Vines are spur-pruned at a bud load of about 10–12 nodes per meter of row. The main cordon wire is located 90 cm from the ground, whereas single foliage wires are placed 30, 70 and 110 cm above the main wire to allow a maximum canopy height of about 2.2 m. Prior to any eventual shoot trimming that might be required to control canopy hindrance, shoots outgrow the top foliage wire by about 30–40 cm and the vineyard fits within a ratio for canopy height-to-width between rows of about 1:1, which, at the specific latitude, is considered optimal to maximize diurnal vineyard light interception while avoiding significant mutual shading between the adjacent rows.

One of the two central rows was selected to run either the light canopy interception readings or the entire canopy transpiration assessment (Fig. S1). The method used to quantify the total amount of daily solar radiation intercepted by the grapevine canopies was based on the approach presented by Giuliani et al.^69^ and Poni et al.^64^, with a few modifications. Light readings were taken via a scanner bar equipped with 64 low-cost phototransistors (BPW20RSilicon PN Photodiode, Vishay Telefunken, Heilbronn, Germany) sensing short-wave radiation in the 300–1100 nm waveband. The sensors were embedded in a lightweight aluminum bar (15 mm wide, 35 mm thick) and spaced at 35 mm to yield a total maximum measuring length of 2205 mm. Each sensor was covered with a Teflon layer to correct the readings in accordance with Lambert’s cosine law. The scanner bar was wired to an externally powered CR10 WP datalogger (Campbell Scientific Ltd, Lough-borough, UK), equipped with an AM 416 relay multiplexer. The data were then expressed as a photon flux density (PFD, μmol photons/m^2^ s), using the multiplying factor of 4.6 reported by Thimijan et al.^70^ for daylight.

Measurements of TCLI were taken on three dates throughout the season: 30 June, 8 July and 18 September, under clear-sky conditions and negligible wind speed. On those dates, the vine canopies were rather stable in terms of volume row filling with negligible leaf area development from the first to the third date. On each occasion, the readings were taken at two-hour intervals between 8 AM and 6 PM. The readings were taken along the test row on three adjacent vines by moving the horizontally held light scanner below the main supporting wire at intervals of 10 cm. This step length allowed us to scan the three vines in about ten minutes (3–4 min per vine). The number of data sets per vine varied between 10 and 20, depending upon the sun position and shape of the canopy shadow, with the total number of measuring points per canopy varying from 640 to 1280. At each measurement, the light scanner was shifted across the row line to include the entire canopy shadow projection (Fig. S1), and incident PFD was retrieved from the same sensor used to monitor incident light for the whole-canopy readings described hereafter. The TCLI per unit of time (μmol photons/s) was calculated as the summation of the difference between the reference (external) incoming light and the value for each pixel of the under-canopy shadow area (μmol photons/m^2^ s) multiplied by the grid area of each pixel (m^2^). Grid area per pixel was, in turn, calculated by multiplying step length (100 mm) by the space between sensors along the scanner (35 mm). Hence, the TCLI could also be presented as a fraction (%) of canopy intercepted light to the total incoming light.

The whole-canopy transpiration (T_c_) was measured using the multi-chamber system described in detail by Poni et al.^71^. In brief, it features alternating current, centrifugal blowers (Vorticent C25/2 M Vortice, Milan, Italy) delivering a maximum airflow of 950 m^3^/h; flexible plastic polyethylene chambers allowing 88% light transmission, 6% diffused light enrichment and no alteration of the light spectrum; a CIRAS-3 DC CO_2_/H_2_O differential gas analyzer (PP-Systems, Amesbury, MA, USA); and a CR1000 data logger wired to an AM16/32B Multiplexer (Campbell Scientific, Shepshed, England).

Along the test row where the light interception readings were taken, six vines were randomly chosen, and the chambers were mounted from 29 June to 10 August and 17 to 21 September, during which the system worked unattended on a 24-h basis. The switching of air sampling between chambers was programmed at 60-s intervals using a set of solenoid valves. The airflow rate fed to the chambers was 13.88 L/s, measured by a constant flow rate diaphragm regulator. Ambient (inlet) air temperature, along with the air temperature at each chamber outlet, was measured by shielded 1- or 0.2-mm-diameter perfluoroalkoxy Teflon insulated type-T thermocouples (Omega Engineering, Stamford, CT, USA). Moreover, direct and diffuse radiations were measured with a BF2 sunshine sensor (Delta-T Devices, Cambridge, England) placed horizontally on top of a support stake next to the chambers enclosing the canopies. Canopy transpiration (T_C_ as mmol H_2_O/s) was assessed from flow rates and H_2_O differentials, and daily T_c_ rates were calculated by averaging instantaneous records taken from dawn until dusk.

At the end of the trial and before the commencement of leaf fall, the six previously chambered vines were entirely defoliated and all the leaves were processed with an automated leaf area meter (LI-COR 3100C, LI-COR, Lincoln, Nebraska, US) to calculate the final leaf area per vine.

### Validation of water stress warnings

In 2021 and 2022, leaf gas exchange and leaf water potential (midday and pre-dawn) were measured in the vineyards coded as V23, V24 and V25 (Table 1) for physiological validation of the water stress warnings released by the Big-VINE app.

The leaf gas exchange was measured through healthy, mid-shoot mature leaves with an LCi-SD (ADC BioScientific Ltd, Hoddesdon, UK) portable gas exchange system featuring a broad-leaf chamber with a 6.25 cm^2^ window. In each season and vineyard, the readings were taken two or three times from mid-June to mid-August. All the readings were taken in the morning (10:00–13:00 solar time) under a clear sky and saturating light conditions at ambient air temperature and CO_2_ concentration on 18 leaves per vineyard × date combination (i.e., two leaves chosen on nine randomly selected vines). Leaf assimilation (A, µmol m^−2^ s^−1^), transpiration (E, mmol m^−2^ s^−1^) and stomatal conductance (g_s_, mol m^−2^ s^−1^) rates were then calculated by the built-in processor from inlet and outlet CO_2_ and H_2_O concentrations, as well as from airflow set at 300 mL/min.

Same leaves used for gas exchange assessment were then measured for midday water potential (ψ_MD_) measured using a Scholander pressure chamber (3500 Model, Soilmoisture Equip. Corp., Santa Barbara, CA). Moreover, pre-dawn leaf water potential (ψ_PD_) was measured at sunrise (∼ 04:00 AM) with the same method, although this time the leaves were sampled from the basal shoot positions.

### Required model inputs and data statistical analysis

Essential inputs required to feed the Big-VINE tool are: site georeferentiation, soil slope, soil analyses as texture and organic matter, row orientation, distance between rows; maximum canopy height, maximum canopy thickness, daily incoming total radiation, daily minimum, mean and maximum air temperature, daily relative humidity, daily wind speed, daily precipitation.

To predict the outcome of a response variable (y) to one or more explanatory variables (x_n_), the multiple linear regression and the nonlinear regression routines from the XLSTAT package (XLSTAT, Addinsoft, Paris) were used. Variation around means was provided as standard error (SE). The Pearson correlation coefficient (*r*) was used to measure strength and direction of linear correlation between two variables.

### Ethical approval

Experimental research and field studies on the cultivated plants, including the collection of plant material here reported, comply with relevant institutional, national, and international guidelines and legislation. Moreover, plant specimens collection was made in line with the appropriate permissions.

## Results

### Data visualization and dashboard

**Figure 3:**
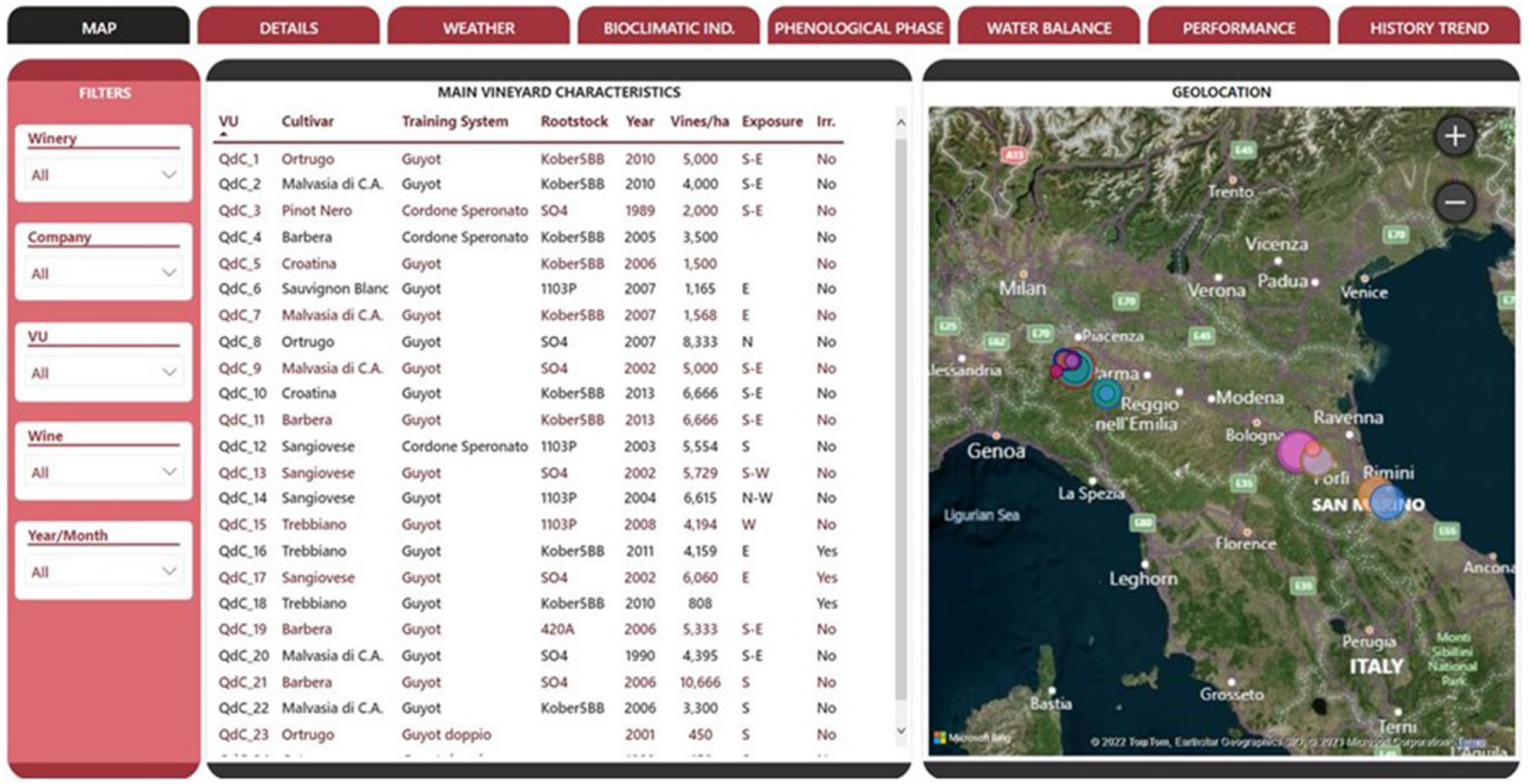
Appearance of the home-page of the Big-VINE app dashboard. Top menu offers navigation through the different items, whereas the filters available on the left allow to select for the specific features. The central body summarizes main features of each vineyard unit (VU) which are shown on the map on the right hand side of the figure. Figure produced with the diagram.net software (Version 21.8.0) freely available at: https://app.diagrams.net/.

The Big-VINE app consists of a series of interactive pages, wherein the various analyses of the data sources are organized and divided. In general, the layout of each page (Fig. 3) consists of a navigation menu (top), allowing to quickly move from one page to another; a menu of filters (left) allowing to easily modify the granularity of the search, starting from a more general view and then focusing on specific cases, and a central body with vineyard features and maps. Besides the navigation and filter tabs, it is also possible to access a tooltip, i.e., an additional, more detailed view of a single piece of information. For instance, the user is easily able to pop up soil analyses values of a specific field (namely texture and organic matter) or, after choosing a given year, to visualize soil water holding capacity at bud burst. When aligning the cursor (when using via the Web) or maintaining pressure (when using via smartphone) on a specific row of the table, a box with additional graphical information is displayed regarding the selected production unit.

### Phenology predictions

**Figure 4:**
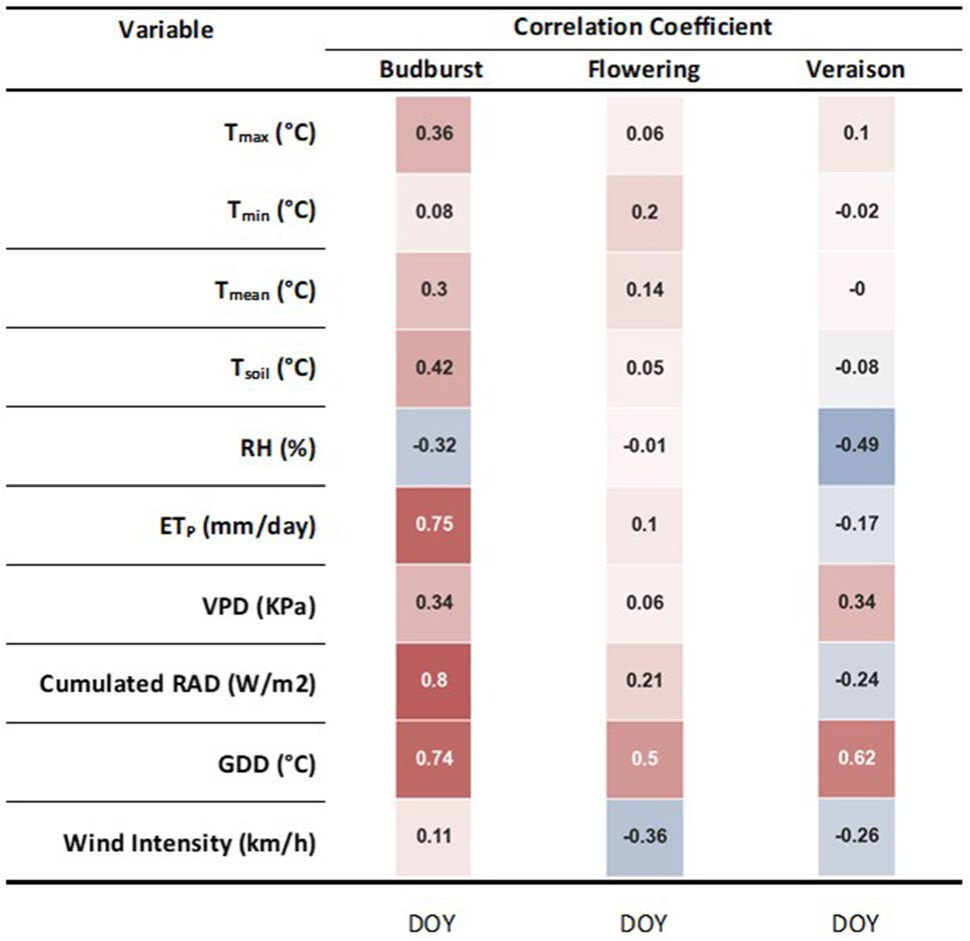
Correlation matrix shown as a heatmap of ten variables vs time (DOY) elapsed between 1 January and date of the observed phenological stage. For each correlation, n = 38. GDD = growing degree days, base 10 °C.

Figure 4 shows the linear correlation coefficient matrix for each phenological stage of the ten climate-based or climate-derived variables against the observed date (DOY) of occurrence. The selection of variables was based on an r > 0.3 or − 0.3 cut-off, which allows us to state that for budburst predictions, ET_P_, cumulated radiation and cumulated growing degree days (GDD) play a key role. A looser correlation was found for T_max_, T_soil_, VPD (positive) and RH (negative). In general, flowering was found to be an unresponsive phenological stage since the cumulative GDD and wind intensity only were above-threshold correlated with the DOY of stage occurrence. Furthermore, veraison was found to have a similar behavior, having cumulated GDD only as a main driver with a minor role for VPD and RH, the latter depicting a negative correlation.

The results of the benchmark for each phenological phase produced for the choice of the best classification algorithm, implying the final prediction, are reported in Table 2. In all benchmarks, the best algorithm was XGBOOST (Extreme Gradient Boosting), leading to an RMSE of 5.6 days for budburst, 2.3 days for flowering and 8.8 days for veraison. Tables S2 and S3 also report, for each year, the observed and predicted dates of budburst, flowering and veraison, as well as heat accumulated (GDD base 10 °C) between 1 January and stage B, for transitions from B to F and between F and V. It was found that coefficient of variation (CV) calculated for data pooled over all the vineyards and years was 70.8%, 12.9% and 19.4% for 1 Jan–B, B–F and F–V, respectively.

**Table 2:** Main metrics for the train, validation and test phases of four different prediction models shown for budburst, flowering and veraison.

Model	Phase	AUC	Accuracy	Recall	Precision	F1-score	RMSE
Budburst							
Random forest	Train	0.988	0.826	1	0.742	0.852	8.21
	Valid	0.808	0.808	1	0.723	0.839	
	Test	0.866	0.593	1	0.064	0.120	
Support vector machine	Train	0.884	0.757	0.978	0.679	0.802	8.80
	Valid	0.735	0.735	0.967	0.661	0.785	
	Test	0.717	0.574	1	0.061	0.115	
K-nearest neighbours	Train	1	0.954	1	0.916	0.956	8.14
	Valid	0.94	0.94	1	0.893	0.943	
	Test	0.762	0.833	0.667	0.105	0.181	
XGBoost	Train	1	0.995	1	0.991	0.995	**5.64**
	Valid	0.971	0.971	1	0.945	0.972	
	Test	**0.876**	**0.944**	**0.667**	**0.286**	**0.400**	
Flowering							
Random forest	Train	0.964	0.799	1	0.501	0.668	3.76
	Valid	0.868	0.789	1	0.491	0.659	
	Test	0.898	0.741	1	0.097	0.177	
Support vector machine	Train	0.847	0.554	0.991	0.311	0.473	4.34
	Valid	0.715	0.554	0.985	0.31	0.472	
	Test	0.825	0.269	1	0.037	0.071	
K-nearest neighbours	Train	0.985	0.954	1	0.816	0.899	2.45
	Valid	0.961	0.937	1	0.764	0.866	
	Test	0.633	0.907	0.333	0.111	0.167	
XGBoost	Train	0.993	0.973	1	0.883	0.938	**2.28**
	Valid	0.975	0.96	1	0.834	0.909	
	Test	**0.813**	**0.935**	**0.333**	**0.167**	**0.222**	
Veraison							
Random forest	Train	0.949	0.583	1	0.545	0.706	8.89
	Valid	0.574	0.574	1	0.54	0.701	
	Test	0.802	0.26	1	0.022	0.043	
Support vector machine	Train	0.754	0.562	1	0.533	0.695	9.24
	Valid	0.561	0.561	1	0.533	0.695	
	Test	0.628	0.22	1	0.02	0.039	
K-nearest neighbours	Train	0.981	0.939	1	0.891	0.942	8.82
	Valid	0.914	0.914	1	0.853	0.921	
	Test	0.688	0.886	0.5	0.071	0.124	
XGBoost	Train	0.994	0.978	1	0.958	0.979	**8.79**
	Valid	0.953	0.953	1	0.915	0.956	
	Test	**0.855**	**0.919**	**0.5**	**0.1**	**0.167**	

RMSE is the standard deviation of the residuals. The best performing model in terms of root mean square error (RMSE) is highlighted in bold. Calculation details of each metric (AUC-ROC, Accuracy, Recall, Precision, and F1-Score) are provided in text.

### Light interception and canopy water use modelling and calibration

**Figure 5:**
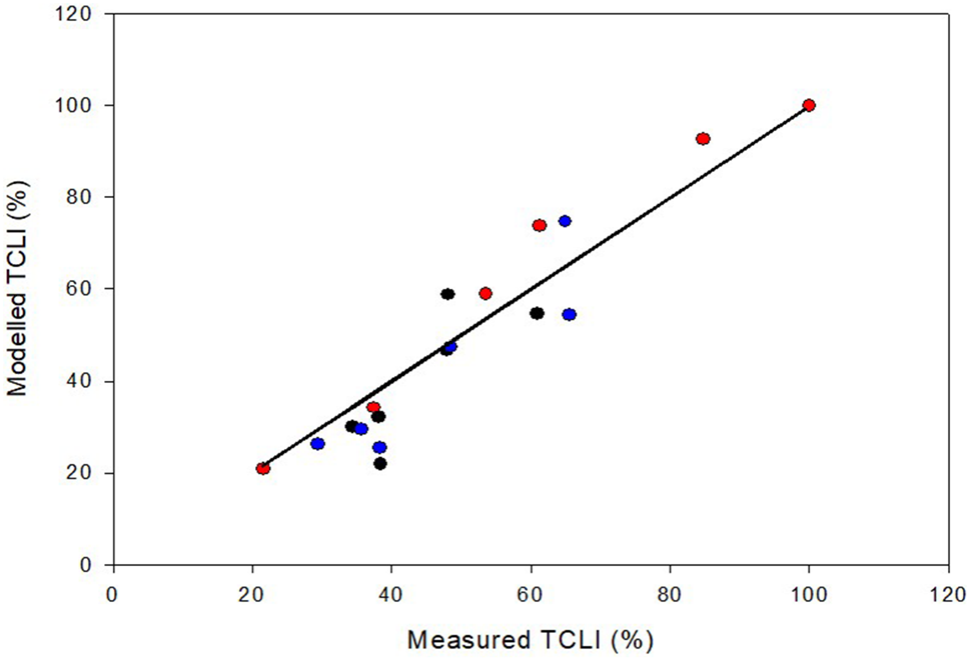
Linear correlation expressed by the Pearson Correlation Coefficient (r) between measured and modelled TCLI for data pooled over three measuring days (30 June—black circles, 8 July—blue circles and 18 September—red circles) and, within each date, over six readings taken at two-hour intervals from 8 AM to 6 PM. Linear equation forced through the origin is: y = 0.9938x, R^2^ = 0.97.

Pooled measurements of TCLI (%) data acquired for three dates (30 June, 8 July and 18 September 2022) at two-hour intervals depicted a very close correlation (R^2^ = 0.96) with the modelled TLCI (Fig. 5). The measured TCLI ranged from about 20% to 100% of incoming radiation, with the lowest values acquired at the time when the sun was at the zenith point (around 2 PM) and the highest (100%) scored by the latest reading on 18 September.

**Figure 6:**
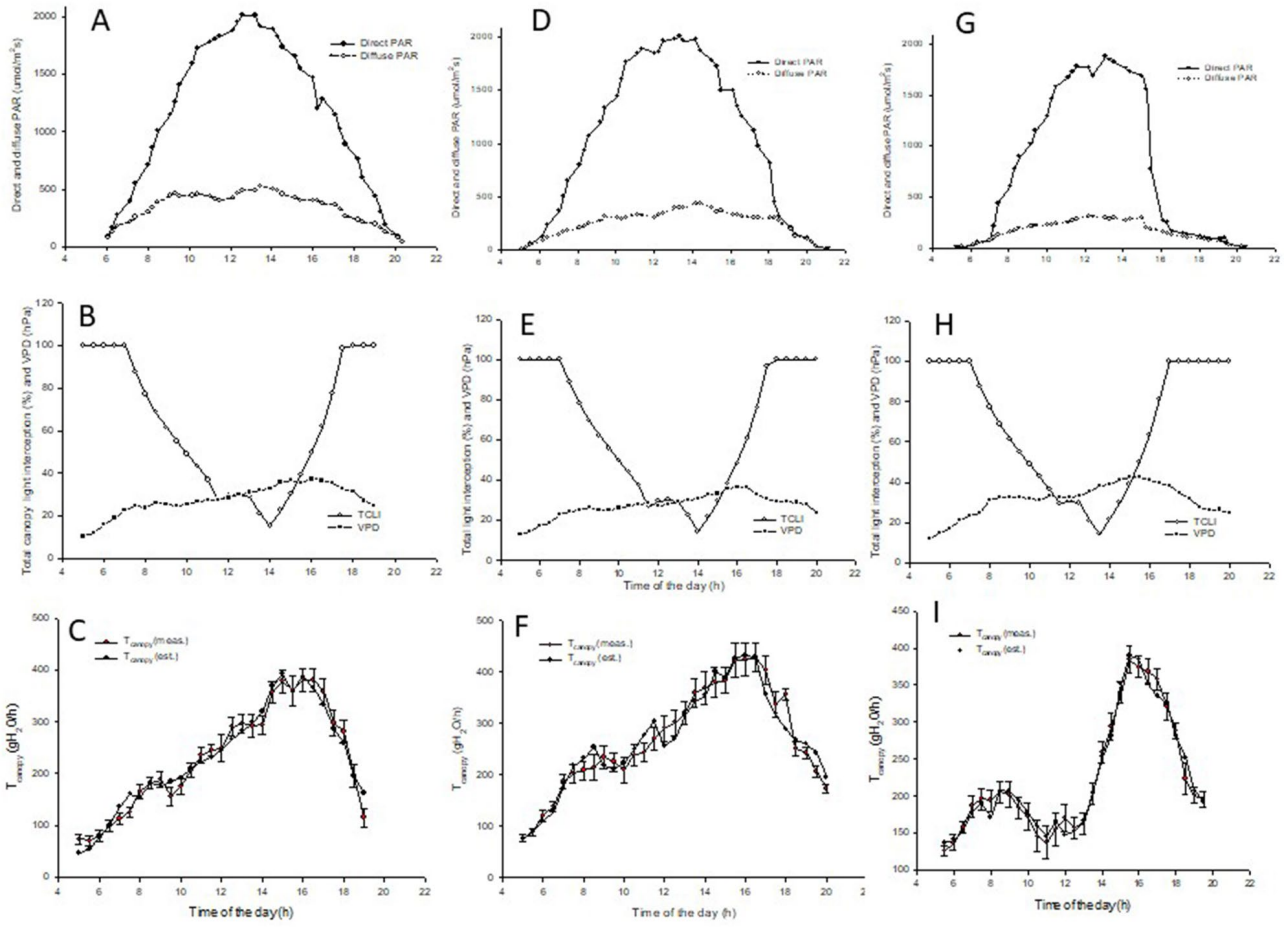
Diurnal trends of direct and diffuse photosynthetic active radiation (PAR) (A,D,G), total canopy light interception (TCLI) and air VPD (hPa) (B,E, H) and measured vs modelled canopy transpiration (T_c_) (C,F,I) for 20 June (left), 2 July (center) and 1 August (right). TCLI is calculated according to equations and vineyard features reported in Table 1. In measured T_c_, each dot is the mean of six vines ± standard error (SE). Modelled T_c_ was obtained through the following non linear (quadratic) multiple regressions: y = 7.62563e−09 × x_1_^2^ + 0.0104358 × x_2_^2^ + 1.49096e−06 × x_3_^2^ +  − 7.72001e−06 × x_1_ × x_2_ + 1.07106e−07 × x_1_ × x_3_ +  − 0.000129436 × x_2_ × x_3_ for 20 June, R^2^ = 0.96; y = − 2.88612e−08 × x_1_^2^ +  − 0.0038447 × x_2_^2^ +  − 3.08502e−06 × x_3_^2^ + 4.12417e−05 × x_1_ × x_2_ + − 7.19364e−07 × x_1_ × x_3_ + 0.00044962 × x_2_ × x_3_ for 2 July, R^2^ = 0.93; y =  − 5.0084e−09 × x_1_^2^ + 0.00716466 × x_2_^2^ + 4.62933e−06 × x_3_^2^ +  − 2.40068e−06 × x_1_ × x_2_ + 1.60601e−07 × x_1_ × x_3_ +  − 0.000160624 × x_1_ × x_2_ for 1 August, R^2^ = 0.98. x_1_ = total direct light; x_2_ = air VPD, x_3_ = TCLI.

Figure 6 depicts the diurnal trends (from dawn to dusk) of direct and diffuse photosynthetic active radiation (PAR), TCLI (%) and air VPD (hPa) and measured and modelled canopy transpiration (T_c_) for three days with clear skies on 20 June (panels A–C), 2 July (panels D–F) and 1 August (panels G–I). Early morning and late afternoon modelled TCLI (%) was set at 100% since at the specific between-row spacing and canopy height and width, canopy shadows extended over the entire alley way spacing (Fig. 6B,E,H). With the sun moving toward higher angles, TCLI progressively decreased with minimum values reached around 1:30–2:00 PM (Figs. 5E,H, 6B and Fig. S2) before recovering in the afternoon hours.

Over the three test days chosen to perform the comparison of measured and modelled T_c_, chamber-derived T_c_ values were consistent in diurnal trends and maximum water use rates. The average canopy water use over the diurnal readings was found to be 229, 268 and 224 g/hour for 20 June, 2 July and 1 August 2022, respectively. Considering the day length for these days (15 h 38 min, 15 h 34 min and 14 h 43 min, respectively), the values equaled 3.58, 4.17 and 3.27 L/vine × day, in the same order. On a per-hectare basis and based on the vine density (4000/ha), the total canopy water consumption ranged between 1.31 and 1.67 mm/ha. As the total leaf area per vine destructively measured at the end of the trial was 3.37 ± 0.018 m^2^, daily canopy transpiration per leaf area unit varied between 0.97 and 1.24 L/m^2^. The measured diurnal trends are apparent on all dates, since a fable morning peak in T_c_ followed by a temporary lag or decrease before progressing towards a second and more pronounced peak occurring around 4 PM were observed.

Under the aim of modelling canopy water use, three potential explanatory (x_n_) variables were considered, i.e., the total amount of direct radiation (µmol m^−2^ s^−1^), TCLI (%) and VPD (kPa). All possible multiple linear and nonlinear regression models employing two or all of the explanatory variables were run to estimate the accuracy of the measured T_c_ over a period of 14 days from 16 June to 10 August. The highest accuracy (R^2^ > 0.88) among all multiple linear and nonlinear regression models was achieved by the quadratic–three variables model with the following equation: y = ax_1_^2^ + bx_2_^2^ + cx_3_^2^ + dx_1_ × x_2_ + ex_1_ × x_3_ + fx_2_ × x_3_, where x_1_ = total direct radiation (µmol m^−2^ s^−1^), x_2_ = air VPD (kPa) and x_3_ = TCLI (%). Among the single explanatory variables used in the multiple non-linear regression model, VPD largely achieved maximum accuracy with an R^2^ value greater than 0.90, whereas the two remaining variables (total direct radiation and TCLI) provided a less accurate and more erratic contribution (Table S4). However, at any date, the accuracy achieved with the multiple non-linear (quadratic) regression approach overwhelmed the accuracy derived from the simple regression equation, which was also observed in the case of VPD. However, the linear regressions conducted on 20 June, 2 July and 1 August for VPD and the measured T_c_ displayed that for VPD values between 0 and 2.5 kPa, the slope of the fitted lines varied from 51.8 and 106.7 mL/h per KPa, whereas for VPD > 2.5 kPa, the slope significantly rose to 155.4 and 209.2 mL/h per KPa.

**Figure 7:**
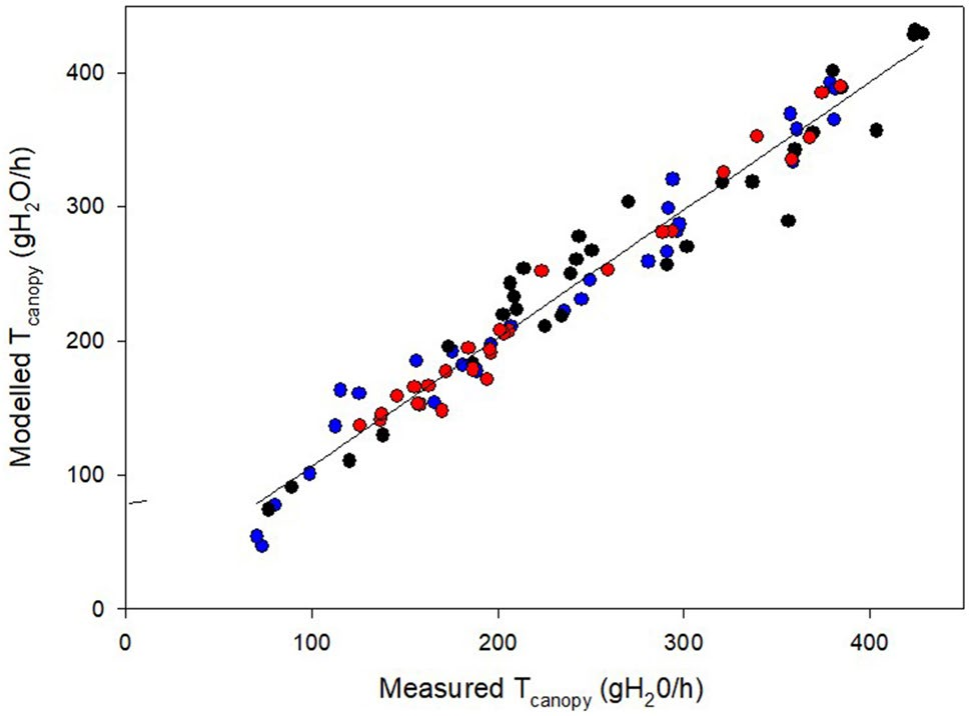
Linear correlation expressed by the Pearson Correlation Coefficient (r) between measured and modelled T_C_ for data pooled over three measuring days (20 June—black circles, 2 July—blue circles and 1 August—red circles) and, within each date, over T_c_ readings recorded at 30 min intervals from dawn to dusk. Linear equation is: y = 0.9938x, R^2^ = 0.97.

Plotting measured and modelled T_c_ together for the three dates confirmed good model sensitivity at tracking T_c_ variation, even on an hourly basis (Fig. 6C,F,I). When measured T_c_ was regressed over modelled T_c_ for the data pooled over the three test days (20 June, 2 July and 1 August), a close linear fit was observed (Fig. 7) following this equation: y = 11.096 + 0.9547x, R^2^ = 0.95.

**Figure 8:**
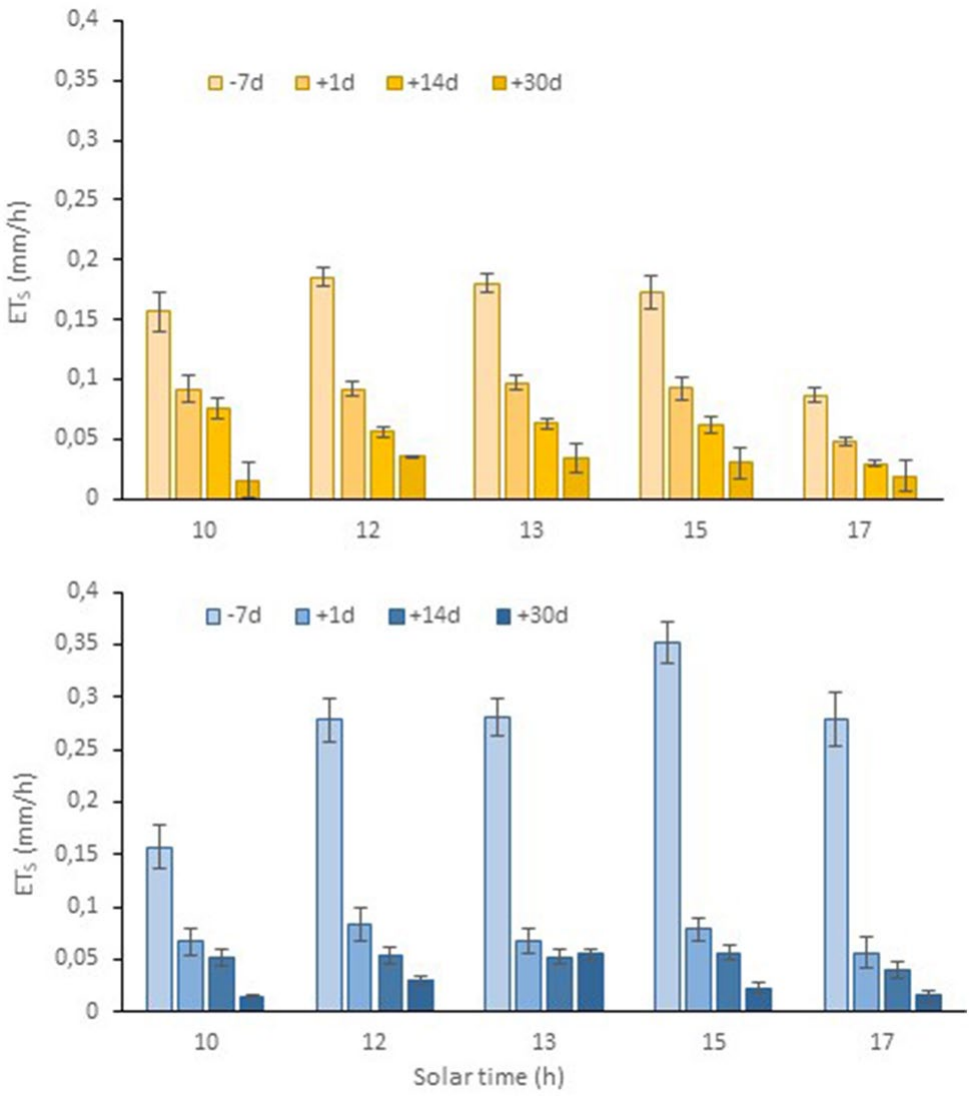
Diurnal soil evapotranspiration rates (ET_S_) measured at the V25 site with a portable closed type system 7 days before and 1, 14 and 30 days after termination of the winter cover crop performed by rolling on 19 May 2022. Top panel refers to the tilled interrow; bottom panel refers to the rolled interrow. Vertical bars represent standard error (SE).

Measurements conducted in 2022 at the V25 site to assess soil contribution, either tilled or grassed, to the vineyard ecosystem water consumption are reported in Fig. 8. Readings taken with the portable closed-type chamber seven days prior to winter cover crop termination indicated a water use of 1.6 ± 0.18 and 2.7 ± 0.22 mm/day in the tilled and cover cropped interrow spots, respectively. When the same reading protocol was repeated the first day after grass termination, ET_s_ in rolled interrow decreased by 26% (2.0 ± 0.17 mm/day), whereas the halving of evaporation rates from bare soil reflected a progressive top-soil drying process. Interestingly, ET_s_ in the rolled alleys dropped by 0.5 ± 0.04 mm/day two weeks post-termination. This amount decreased further a month after termination. The overall data set gathered on diurnal and seasonal soil water use of the two treatments allowed us to establish a preliminary trend of soil water loss that strictly referred to the canopy. During wintertime, under the combination of no canopy and low temperatures, a constant baseline of 0.3 mm/day of water use was used.

### Drought warnings and validation

**Figure 9:**
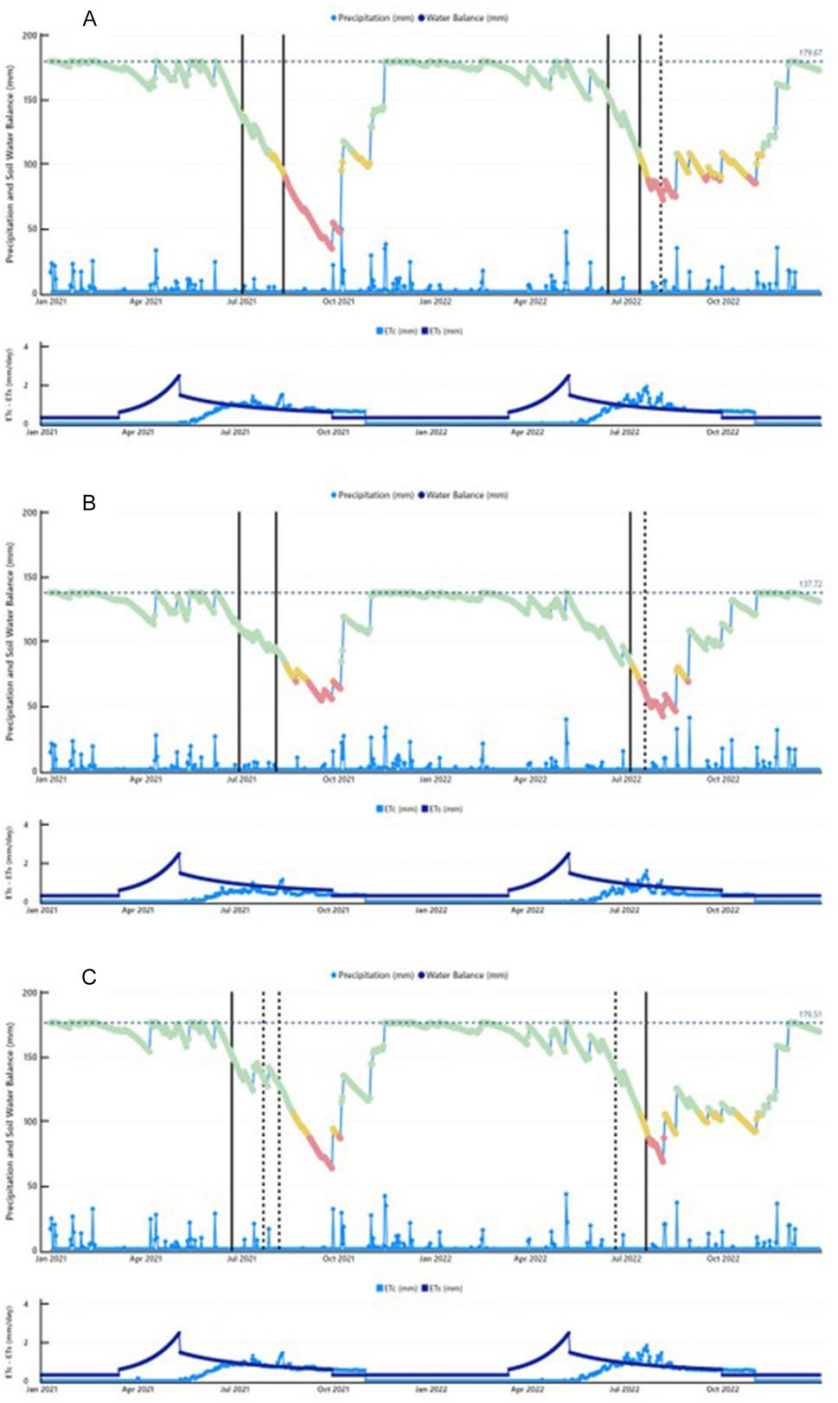
Seasonal water balance and daily precipitation (top panel) and soil evapo-transpiration (ET_s_) and canopy transpiration (T_c_) (bottom panel) measured and or estimated for 2021 and 2022 at V23 (**A**), V24 (**B**) and V25 (**C**). In each graph the dotted horizontal line indicates the value of field capacity calculated from texture and organic matter content according to^99^. Vertical solid lines identify dates with a correct match between drought warning release and in situ—gas exchange assessment; vertical dotted lines identify false-negative and false positive cases. Estimated ETs is based on actual cover crop and soil ET rates taken at V25 under the configuration of permanent tillage under the row and temporary winter crop between rows then terminated in spring through rolling. The same soil management strategy was adopted in the other vineyard units.

The three vineyards coded as V23, V24 and V25 were modelled in 2021 and 2022 for estimates of the seasonal water balance (canopy and soil contributions) and consequent drought warnings, which were validated versus leaf gas exchange and water status readings taken two or three times in each respective summer season (Fig. 9A–C; Table 3). The total number of warnings released for the data pooled over the years and the vineyard units was found to be 14. In the V23 unit, four out of the five drought warnings released were a perfect match (Fig. 9A). A slight mismatch was observed for the third sampling in 2022 (DOY 215), when the model depicted red code, whereas the leaf sampling, performed the same day indicated mild stress (A = 6.35 µmol m^−2^ s^−1^ and Ψ_PD_ = − 4.63 bars). In the V24 unit, both leaf samplings made on DOY 180 and 215 agreed with the model warnings, depicting no relevant stress (green code). However, in 2022, a correct matching (no stress) was seen on the first date of validation (DOY 186, 5 July), whereas the model gauged a false positive on DOY 200 (July), when a red code was reflected by a quite mild, initial stress according to single leaf sampling (Ψ_PD_ = − 4.63 bars). For the V25 unit, in 2021, the first sampling date correctly confirmed a no-stress status (green code), whereas, for the remaining two sampling dates (DOY 203 and 2018), the model resulted in two false negatives, as the gas exchange readings highlighted mild and severe stress, respectively. Another false negative was observed upon the first validation reading in 2022, whereas a correct match was found for the red alert delivered on DOY 201. Overall, out of a total of 14 validation events, nine were correct, two resulted in false positives and three resulted in false negatives.

**Table 3:** Mean leaf transpiration (E, mmol m^−2^ s^−1^), stomatal conductance (g_s_, mmol m^−2^ s^−1^), assimilation (A, µmol m^−2^ s^−1^) rates and midday leaf water potential (Ψ_md_, bar) and pre-dawn leaf water potential (Ψ_pd_, bar) values recorded in 2021 and 2022 on single mature leaf samples (n = 18) and then used for validation of modeled water stress alarms.

Vineyard code	Year	DOY	E (mmol m^−2^ s^−1^)	g_s_ (mmol m^−2^ s^−1^)	A (µmol m^−2^ s^−1^)	Ψ_MD_ (bars)	Ψ_PD_ (bars)	VPD (kPa)
V23	2021	183	4.96 ± 0.41	0.23 ± 0.03	9.73 ± 0.61	− 9.68 ± 0.12	− 2.37 ± 0.51	1.22
		222	2.76 ± 0.22	0.06 ± 0.01	5.69 ± 0.97	− 11.6 ± 0.64	− 4.28 ± 0.42	1.66
	2022	165	4.55 ± 0.22	0.16 ± 0.01	14.25 ± 0.78	− 9.72 ± 0.43	− 1.92 ± 0.11	1.07
		195	2.88 ± 0.45	0.05 ± 0.01	4.15 ± 1.00	− 11.13 ± 0.1	− 4.87 ± 0.35	2.15
		215	3.96 ± 0.26	0.08 ± 0.01	6.35 ± 0.72	− 13.11 ± 0.64	− 4.63 ± 0.42	1.82
V24	2021	180	5.45 ± 0.35	0.19 ± 0.02	10.78 ± 0.61	− 8.02 ± 0.07	− 2.00 ± 0.04	1.28
		215	3.69 ± 0.18	0.14 ± 0.01	12.5 ± 0.69	− 10.02 ± 0.27	− 3.83 ± 0.21	0.60
	2022	186	5.71 ± 0.24	0.21 ± 0.01	11.84 ± 0.73	− 11.08 ± 0.33	− 3.83 ± 0.42	1.61
		200	5.53 ± 0.35	0.13 ± 0.01	10.7 ± 0.92	− 11.83 ± 0.58	− 4.33 ± 0.22	2.17
V25	2021	173	5.20 ± 0.30	0.15 ± 0.02	9.05 ± 0.54	− 10.83 ± 0.14	− 2.68 ± 0.09	1.20
		203	1.96 ± 0.17	0.04 ± 0.01	4.78 ± 0.55	− 14.13 ± 0.12	− 5.76 ± 0.35	1.25
		218	1.84 ± 1.84	0.03 ± 0.003	3.43 ± 0.40	− 14.3 ± 0.14	− 7.06 ± 0.28	1.08
	2022	172	2.9 ± 0.44	0.05 ± 0.01	4.27 ± 0.73	− 13.08 ± 0.35	− 4.9 ± 0.19	1.54
		201	1.14 ± 0.32	0.02 ± 0.004	1.41 ± 0.48	− 14.26 ± 0.24	− 8.03 ± 0.53	2.36

Features of V23, V23 and v25 vineyard units are reported in Table S1. Except for VPD, data are means ± SE. VPD is the mean calculated over the time window (10:00–13:00) during which the gas exchange readings were taken.

## Discussion

Complexity of constructing an effective learning dashboard visualizations is comprehensively discussed in Sedrakyan et al.^72^. Dashboard reported in Fig. 3 reunites six different tasks: overview, zoom, filter, relationship, history and extract in order to reach the following goals: (i) clearly illustrates the point: conveys the intended information with sufficient level of detail, does not contain redundant/irrelevant information; (ii) adapted to the intended audience. This item is crucial in the viticulture realm where an especially user friendly approach is needed due average to growers’ age and scant familiarity with digital tools^73^; (iii) are memorable to those who care about the material and (iv) makes an impact which increases the understanding of the subject matter.

### Phenology predictions

The prediction of the occurrence of budburst, flowering and veraison stages was conducted as per the binary event classification paradigm^57^, which is an original approach when compared to the more traditional modelling followed in previous studies^74–77^. In terms of the sensitivity of the budburst stage to the ten examined variables, our results confirm that cumulated GDD, as well as linked variables including cumulated radiation and ET_p_, are good drivers of the budburst date, as also shown in previous studies^8,75,78^. However, our calibration conditions (i.e., 22 vineyards spread along the Emilia Romagna regions assessed over two seasons) ascertained that the swollen bud stage can be reached across a wide range of GDD calculated from January 1 until the date of the observed event (5.5 to 47.5 °C with a CV of 70.8%). This is physiologically significant and suggests a nonlinear relationship between heat summation and bud break occurrence. In this context, Sadras and Moran^79^ proposed that the thermal effects on phenology are modulated by the interplay between resource-driven growth and temperature-driven development. They also suggested that a thermal effect on phenology progression can be enhanced if the vine also bears a high leaf area-to-fruit ratio. Moreover, the prediction accuracy for the budburst date significantly improved when traditional GDD was associated, for instance, with an index taking into account the length of the winter dormancy^77^. The results of our study appear a bit alarming for growers as the attainment of the swollen bud stage (and hence, the stage beyond which susceptibility to spring frost damage raises exponentially) might occur even at low GDD values, therefore invalidating forecast methodologies based on the principle that a given minimum heat load must be reached to enter bud swelling.

In our study, the prediction accuracy for bud burst given as RMSE was 5.6 days with the help of the test phase of the XGBoost model. This was slightly higher than the range of RMSE acceptance, which, according to Yang et al.^80^, is defined as between two to four days for grapevine phenology. However, it should be noted that this precision is already satisfactory if the limited data set used for direct phenology assessment (22 vineyards × 2 years) is considered. Notably, an end user, who eventually owns a more robust data record (i.e., ten years of phenology assessment), might reasonably aspire to acquire rewarding accuracy.

The modeling of the flowering date suggested that this stage was sensitive only to GDD (r = 0.50); however, on the other side, prediction accuracy was reached again through the XGBoost model and proved to be quite good (RMSE = 2.3). According to Reis et al.^81^, the predictive capacity of the models for budburst remains lower than those for flowering and veraison models; however, flowering date prediction in the grapevine must deal with at least two peculiar traits: (i) pronounced scalarity of flowering within the vineyard, vine and cluster, which is likely added to the need of providing a quantitative visual estimate of open flowers to total and impacting the reliability of a by-eye assessment; and (ii) the grapevine owning an internal biological clock^82,83^, according to which the flowering stage is approached when the total number of unfolded leaves on the main shoot is around 16–17. This seems to occur across varieties and vigor levels and can be explained in simple terms as the change in internode length while the leaf number stays constant. It cannot be ruled out that such a trait renders flowering less sensitive to genetic-related variability and climate conditions, which, in turn, influence vine vigor, especially in the time window comprised between budburst and flowering.

The prediction for veraison confirmed the main role of cumulative GDD (r = 0.62) and, more surprisingly, RH (r = − 0.49). The best accuracy of prediction of the onset of veraison was reached by the RGBoost model with an RMSE value of 8.8 days, which was somewhat less accurate as compared to previous works^84,85^. For veraison, the criteria selected to state that the stage has been reached appears to be deceiving; in our work, softening was chosen for determining the veraison date which usually anticipates the onset of color by a few days^16^. Therefore, using deformability or sight-perceivable color appearance might indeed impact the observed veraison date. It also appears a little peculiar that air RH is also involved in determining veraison timing; however, the combined action of air temperature and RH on the VPD has been shown to deeply modify berry transpiration, which, in turn, affects net sugar intake by the berry^86^ as well as the ripening speed^87^.

The modeling approach used in this study with the ambition to sense incoming water stress based on standard weather and soil data and a few simple vineyard characteristics was founded on two strong pillars: robust calibration of modelled TCLI vs measured light interception and an equally good calibration of modelled canopy water loss rates vs measured whole-canopy transpiration. As shown in Figs. 5 and 7, both pillars were successfully achieved. Regarding the former calibration, to warrant the highest accuracy, we also wished to examine how the daily water consumption by a canopy is composed during the day and how this relates to the diurnal variation in TCLI. A visual comparison between diurnal trends of TCLI and T_c_ reported for June 20, July 2 and August 1 (Fig. 6) reveals an apparent mismatch. For instance, under the specific site location and row orientation, when the sun is at the zenith point (around 2 PM) and the amount of TCLI is at the minimum (i.e., less than 20% of incoming), the measured T_c_ is still quite high, setting at about 80% of maximum transpiration loss reached two hours later (about 4 PM) and quantified at about 400 mL/h. This is also confirmed by linear correlation coefficients calculated on each day over diurnal values of TLCI and T_c_, which varied between 0.45 and 0.42. Such consistent diurnal patterns apparently contradicted the quite shared principle that grapevine water use is a close linear function of the shaded area measured underneath the canopy^62^. However, calibration provided in the latter study exploited a seasonal variation in the leaf area per vine from 2 to 34 m^2^. Moreover, to allow even more variability, both curtains of the trellis were raised for a couple of weeks to resemble an overhead pergola, thereby increasing light interception. However, this did not occur in the current study. We worked on static canopies (around 3.0–3.5 m^2^ of leaf area), and the TCLI varied due to the interaction between sun position, canopy geometry and row orientation. Therefore, concluding that the canopy water use is the least at 2 PM simply because TCLI is at its minimum would be wrong. In addition, a previous work where whole-canopy transpiration was tracked during the day showed that under a perfect NS orientation, a temporary gap in transpiration can be observed during the central hours of the day, which tracks lower TCLI at that time^88^. Using a similar approach, Buesa et al.^89^ found that at the latitude of Valencia (Spain), the seasonal canopy water use by an EW-oriented row was 18% less than the one measured on an NS-oriented row. It is inherent that a model that can include diurnal variability in its TCLI estimation due to row orientation or soil slope is more accurate than any other approach, considering an average daily TCLI estimated from a quite impersonal crop coefficient.

Moreover, of the three variables taken into consideration to predict T_c_ in our study, VPD was largely predominant, whereas total direct light and T_c_ depicted poor correlation (r varying between 0.18 and 0.20). In a previous study where a light saturation curve for whole-canopy transpiration was assessed on NS-oriented five-year-old Chardonnay grapevines with a total leaf area between 3.4 and 4.8 m^2^^88^, T_c_ peaked at a PFD of about 1200 µmol m^−2^ s^−1^, whereas due to more light directly lost onto the ground, PFD values beyond that threshold resulted in decreasing T_c_. Panels B, E and H of Fig. 6 depict that for the two times during the day (morning and afternoon) when direct PAR transits across the 1200 µmol m^−2^ s^−1^ threshold, the corresponding modelled TCLI is quite similar, whereas the T_c_ measured in the afternoon time window is double that measured in the morning time window. VPD is clearly responsible for this scenario, and notably, on any of the test days, the slope of the T_c_ increase per KPa unit significantly increased beyond the 2.5 kPa threshold.

Stomatal responsiveness to evaporative demand (air VPD) varies widely between *Vitis vinifera* L. cultivars, and mechanisms for stomatal control in response to VPD remain quite obscure, especially regarding differential stomatal sensitivity of iso vs aniso-hydric genotypes^90,91^. Indeed, the response observed in our study on Cabernet Sauvignon under well-watered conditions would suggest that transpiration dragged by high VPD afternoon values is the main determinant of the decreasing response of instantaneous water use efficiency (WUE_inst_) to leaf-to-air VPD observed on the four grapevine varieties (i.e., Silvaner, Syrah, Grenache, Airen), which are grown in pots over a wide range of climate conditions, e.g., PFD between 750 and 1750 µmol m^−2^ s^−1^; air temperature varying between 19 and 37 °C; and conditions of different drought and recovery experiments; and pre-dawn soil water potential comprised between − 0.15 to − 1.45 MPa, as reported by^92^. Moreover, the chance of canopy water loss rates increasing above a VPD threshold of ~ 2.5 kPa is quite alarming if current vs historical and projected climate trends are taken into account^93,94^. A constant feature noted in several grape growing regions across Europe^95^ is that over the three summer months of June, July and August, the potential evapotranspiration (ET_P_) has increased over the last two decades as compared to previous historical series. In Bordeaux such an increase was about 100–120 mm as compared to the 1951–1990 period^1^, whilst a similar trend has been reported for the Oltrepò Pavese Region in Northern Italy^96^. These trends also justify why a so-called “meteorological drought” can set in without necessarily having limiting conditions in soil moisture^96^.

### Drought warnings

A primary objective of this study was to assess the preliminary reliability of a warning system for incoming water stress which uses the vines as a main proxy (i.e., no sensors are needed for soil or plant water status monitoring) and with the feeding of the elementary vineyard, climate and soil data. To the best of our knowledge, only one other study applied a similar approach^25^. It was a warning system working at a minute scale on discontinuous canopies with warnings tailored to specific vineyard characteristics. In this approach, while satisfactory calibration was provided on Barbera vines trained according to two different pruning systems (cane pruned and spur pruned), validation (i.e., the process of confirming that the model actually achieves its intended purpose under conditions that differ from the calibration environment) was missing.

The validation carried out in three different vineyards over two consecutive seasons in our study yielded a 64% success, which should be considered an encouraging start. Optimization of the alarming system may be pursued from various angles; albeit analyzing possible causes of false positives and negatives is necessary. To start with the two false positives marked by the warning system, the combination of V23-2022-DOY215 depicting a red alert when the gas exchange assessment indicates moderate stress is a tenuous mismatch. While the 12.2 mm of precipitation that occurred over the week preceding the readings was still insufficient to allow SAW to re-enter a yellow code area, rainwater reaching shallow roots along with a day marked by average mean VPD (1.82 kPa) had probably begun the replenishment of leaf function. A similar explanation seems to hold for the false positive detected on V24-2022-DOY200 (red alert vs. moderate stress based on leaf gas exchange), although a lack of precipitation during the 20 days preceding the assessment seems to pose a more challenging case. A yellow or red alert, which is not confirmed through either visual scouting or by physiological validation, might have different causes among which, especially when facing mature vineyards established on rather deep soils as those included in our sample, having some tap roots digging underneath the default soil depth of 1 m and assuring some precious water uptake^97^ is a likely hypothesis. However, the Big-VINE app boasts a good interaction with the end-user, who can enter a deeper maximum soil depth in the algorithm, which will clear the alarm until a new warning is released.

In our study, the validation procedure of the water stress warnings also highlighted three false negative cases, which exclusively occurred at the V25 unit (Table 3). This suggests that true water availability was likely to be less than the initial soil reservoir calculated by the model. Mirroring the solution envisaged for the previous case, we suspect that the actual soil depth was shallower than the assigned 1 m values. However, a previous work conducted in the same vineyard where the soil profile was studied^98^ showed that a good homogeneous soil is found until 2 m depth, rendering unlikely the hypothesis of root growth restriction. Hence, attention should be devoted to any factor that might restrict root water uptake, which can be negatively influenced by an overcropping status coupled with low soil fertility. The data reported by Gatti et al.^98^ for the same vineyard unit confirms a general source limitation (leaf area-to-yield ratio between 0.51 and 0.68 m^2^ kg^−1^, a low organic matter content [1.2% between 0 and 60 cm] and a scarce total N content [0.64‰]).

## Conclusions

A new online Big Data based tool named Big-VINE exclusively relying upon elementary inputs of the vineyard ecosystem and standard weather and soil data, was tested in terms of prediction accuracy for key phenological stages and actual meteorological drought in vineyards. Although a still quite small data set was used, the binary event classification paradigm utilized to assess the Machine Learning algorithms was able to provide fair and good prediction accuracy for budburst and flowering, respectively. The released drought warnings, assessed by comparing them to in situ readings of leaf gas exchange and water status, were found to be correct in 9 out of a total of 14 case studies. This seems a remarkable outcome if the simplicity of the requested input is considered and due to the fact that Big VINE does not require the implementation of any fixed bulky and expensive sensor. It can thus be concluded that Big-VINE can valuably assist growers under a condition of either occasional or ordinary drought. In the former case (i.e., premium wine regions in Central-Northern Italy and France), where irrigation is still not the rule and is even skeptically regarded, growers require guidance through a user-friendly approach that helps them to avoid major mistakes, such as anticipated or excessive irrigation leading to a deleterious forcing technique or late interventions, often based on tardy visual assessment. Hence, if the specific environment configures the condition of ordinary irrigation, Big-VINE can furnish an instantaneous output for soil plus canopy water use, which helps setting the water supply. Future outlook of Big VINE encompasses the introduction of sub-routines which might better deal with the role that a given soil management strategy has on seasonal water use and it will also expand to include modelling of net whole-canopy CO_2_ assimilation with the aim of alerting about possible source limitations occurring at critical growth and ripening stages.

### Supplementary Information

Supplementary Figures.

Supplementary Table S1.

Supplementary Tables.

Supplementary Table S4.

## Data Availability

All data generated or analyzed during this study are included in this published article and its Supplementary Information files.
