# Supplementary material for: A sensorless, Big Data based approach for phenology and meteorological drought forecasting in vineyards
Source: Sci Rep. 2023 Oct 5;13:16818. doi: 10.1038/s41598-023-44019-4 (PMC10556084; doi:10.1038/s41598-023-44019-4)
Supplement: Supplementary file 1 — Supplementary Figures. [file 41598_2023_44019_MOESM1_ESM.pptx]

## Slide 1
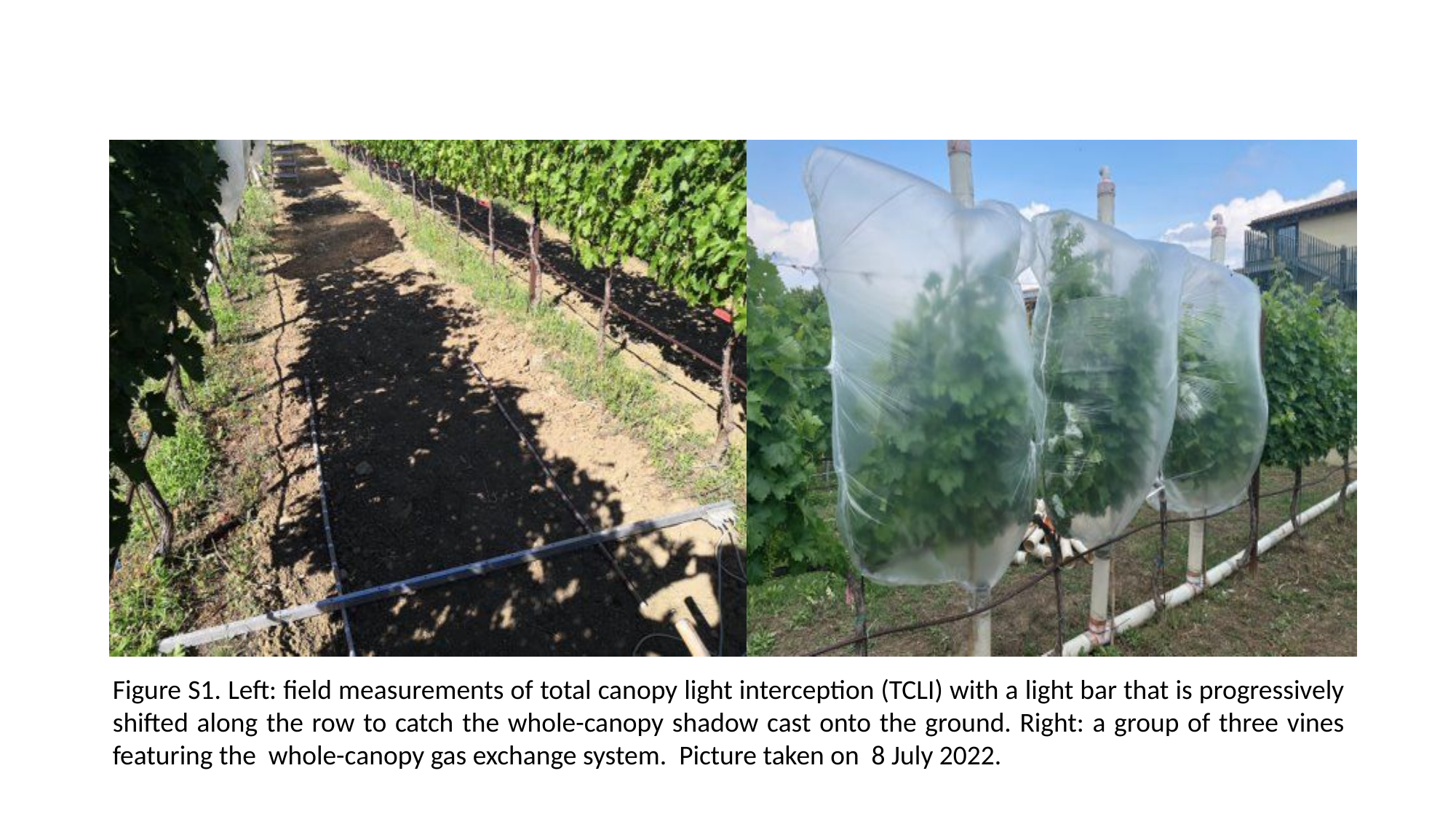

Figure S1. Left: field measurements of total canopy light interception (TCLI) with a light bar that is progressively shifted along the row to catch the whole-canopy shadow cast onto the ground. Right: a group of three vines featuring the whole-canopy gas exchange system. Picture taken on 8 July 2022.
