# Supplementary material for: A sensorless, Big Data based approach for phenology and meteorological drought forecasting in vineyards
Source: Sci Rep. 2023 Oct 5;13:16818. doi: 10.1038/s41598-023-44019-4 (PMC10556084; doi:10.1038/s41598-023-44019-4)
Supplement: Supplementary file 2 — Supplementary Table S1. [file 41598_2023_44019_MOESM2_ESM.docx]

**Table S1.** Variables, equations and calculations performed for determination of solar radiation components hitting an horizontal surface and an hedgerow trained vineyard row. Simulation is performed for 20 July (DOY 201) 2022 at the Piacenza location (Northern Italy). Reworked from Giuliani et al.(2016), Pollet et al. (2005) and Wong and Chow (2001).

| **Parameter** | **Variables and range (if any)** | **Abbreviation and unit** | **Value** | **Notes** |
| --- | --- | --- | --- | --- |
| **Air physical conditions** | Condensable water | HE, cm | 1 |  |
| “ | Turbidity coefficient, 0-0.01 | Dimensionless | 0.01 |  |
| **Coordinates in space and time** | Latitude | La, degrees | 45.05 |  |
| “ | Longitude | Lg, degrees | 9.60 |  |
| “ | Time zone | Fo | -1 |  |
| “ | Solar/legal time, 0 or 1 | Le | 0 |  |
| “ | Julian day (0 to 365) | J, number | 201 (20 July 2022) |  |
| “ | Time of day (0-24; 0-60) | Hour, min | 2 PM |  |
| **Coordinates of the exposed surface** | Inclination, 0 = vertical; 90 = horizontal | HV, degrees | 0 |  |
| “ | Orientation, 90 = South; 0 = East | AV, degrees | 125 | Reference to a 35° NE-SO exposed vineyard |
| **Vineyard characteristics** | Vineyard surface | Vs, m^2^ | 10,000 |  |
| **“** | Number of rows | Nr (100/2.5), number | 40 |  |
| **“** | Canopy width | Cw, m | 0.35 |  |
| **“** | Canopy height | Ch, m | 1.20 |  |
| **“** | Surface width | Sw(nr*cw*100)/vs | 0.14 |  |
| **“** | Surface height | Sh (nr*ch*100)/vs | 0.48 |  |
| **Instantaneous calculations** | Time conversion | T, hours | 14 | Instantaneous results at 2 PM |
| **“** | Time equation | oET(J) * 60, min | - 5.948 |  |
| **“** | Hour angle | oAH(t, Fo, Le, Lg, J) * 180/12, degrees | 23.14 | Solar angle vs solar noon |
| **“** | Declination | gD(J), degrees | 20.951 |  |
| **“** | Solar azimuth | AS (t, Fo, Le, Lg, J), degrees | 34.847 |  |
| **“** | Meridian height | HS (t, Fo, Le, Lg, J), degrees | 59.295 |  |
| **“** | Solar radiation angle to the horizontal surface | HSM (t, Fo, Le, Lg, J), degrees | 64.016 |  |
| **“** | Cosine direction | Cos α (t, Fo, Le, Lg, J, AV,HV), degrees  Cos β (t, Fo, Le, Lg, J, AV,HV), degrees  Cos ϒ (t, Fo, Le, Lg, J, AV,HV), degrees | 29.256  -49.264  -0.078 |  |
| **“** | Energy outside the atmosphere | EJ (J), Watt /m^-2^ | 1.32*10^3^ |  |
| **“** | Unit air mass | A(t,Fo, Le, Lg, La, J), number | 0.072 |  |
| **“** | Air mass crossed | M(t,Fo, Le, Lg, La, J), number | 1.162 |  |
| **“** | Turbidity | T(t,Fo, Le, Lg, La, J), number | 2.446 |  |
| **“** | Radiation normal to the surface | ESn (t,Fo, Le, Lg, La, J), Watt /m^-2^ | 1.069*10^3^ |  |
| **“** | Direct radiation | ES (t, Fo, Le, Lg, La, J, AV, HV, Ss, Sv) Watt/m-^2^ | 307.41 |  |
| **“** | Diffuse radiation | Edf (t, Fo, Le, Lg, La, J, Ss, Sv), Watt /m^-2^ | 247.501 |  |
| **“** | Direct plus diffuse radiation | (ES + Edf), Watt/ m^-2^ | 554.911 |  |
