# Supplementary material for: A sensorless, Big Data based approach for phenology and meteorological drought forecasting in vineyards
Source: Sci Rep. 2023 Oct 5;13:16818. doi: 10.1038/s41598-023-44019-4 (PMC10556084; doi:10.1038/s41598-023-44019-4)
Supplement: Supplementary file 3 — Supplementary Tables. [file 41598_2023_44019_MOESM3_ESM.docx]

Table S2. Observed and forecast dates (DOY) for bud burst (B), flowering (F) and veraison (V) recorded in 2021 at each of the monitored vineyard unit (VU). GDD = growing degree days base 10°C cumulated from 1 Jan -stage B, stage B-F and stage F-I. Rows in bold character identify vineyard unit used as test data set.

| **2021 phenology predictions** | | | | | | | | | |  |
| --- | --- | --- | --- | --- | --- | --- | --- | --- | --- | --- |
| **VU** | **Obs.​** | **GDD Jan1-B** | **Forecast**​ | **Obs.**​ | **GDD**  **B-F** | **Forecast**​ | **Obs.**​ | **GDD F-V** | **Forecast**​ |  |
|  |  |  |  |  |  |  |  |  |  |  |
| 1​ | 74​ | 5.6 | 88 | 155 | 208.1 | 154 | 218 | 752.1 | 206 |  |
| 2​ | 74 | 5.6 | 88 | 158 | 235.1 | 158 | 209 | 621.9 | 204 |  |
| 3​ | 79 | 5.6 | 86 | 153 | 191.8 | 153 | 206 | 628 | 204 |  |
| 4​ | 88 | 13.5 | 88 | 158 | 256.4 | 158 | 213 | 716.2 | 227 |  |
| 5​ | 98 | 47.5 | 88 | 158 | 222.4 | 158 | 221 | 808.3 | 227 |  |
| **6**​ | **98** | **47.5** | **88** | **160** | **240** | **157** | **213** | **698.6** | **209** |  |
| 7​ | 88 | 13.5 | 88 | 161 | 285 | 161 | 215 | 709.2 | 229 |  |
| 8​ | 92 | 29.7 | 88 | 162 | 228.6 | 162 | 222 | 715.4 | 203 |  |
| 9​ | 88 | 8.3 | 88 | 161 | 239.9 | 161 | 213 | 630.4 | 205 |  |
| **10**​ | **98** | **37.6** | **88** | **164** | **241.9** | **160** | **230** | **813.6** | **214** |  |
| 11​ | 92 | 29.7 | 91 | 160 | 208.3 | 160 | 214 | 651.4 | 214 |  |
| 12​ | 68 | 8.8 | 90 | 144 | 233 | 144 | 205 | 785.7 | 211 |  |
| 13​ | 72 | 9.7 | 90 | 143 | 220.1 | 143 | 199 | 700.2 | 222 |  |
| 14​ | 87 | 14.7 | 87 | 159 | 338.9 | 144 | 226 | 980 | 226 |  |
| 15​ | 76 | 7.9 | 84 | 151 | 265.6 | 151 | 223 | 1008.7 | 225 |  |
| 16​ | 71 | 10.2 | 74 | 148 | 266 | 148 | 215 | 939 | 226 |  |
| 17​ | 70 | 10.2 | 71 | 146 | 251.2 | 146 | 204 | 771.8 | 226 |  |
| 18​ | 77 | 13.2 | 71 | 153 | 312 | 149 | 216 | 939.6 | 216 |  |
| **19**​ | **91** | **31.3** | **91** | **163** | **278.8** | **163** | **231** | **881** | **208** |  |
| 20​ | 91 | 31.3 | 91 | 163 | 278.9 | 163 | 218 | 694 | 208 |  |
| 21​ | 91 | 22.7 | 91 | 163 | 251.6 | 163 | 227 | 800.5 | 227 |  |
| 22​ | 91 | 22.7 | 91 | 163 | 251.6 | 163 | 227 | 800.5 | 227 |  |

Table S3. Observed and forecast dates (DOY) for bud burst (B), flowering (F) and veraison (V) recorded in 2022 at each of the monitored vineyard unit (VU). GDD = growing degree days base 10°C cumulated from 1 Jan -stage B, stage B-F and stage F-I. Rows in bold character identify VU used as test data set.

| **2022 phenology predictions** | | | | | | | | | |  |
| --- | --- | --- | --- | --- | --- | --- | --- | --- | --- | --- |
| **VU** | **Obs.​** | **GDD Jan1-B** | **Forecast**​ | **Obs.**​ | **GDD**  **B-F** | **Forecast**​ | **Obs.**​ | **GDD F-V** | **Forecast**​ |  |
|  |  |  |  |  |  |  |  |  |  |  |
| 1​ | 86 | 9.2 | 85 | 145 | 248.3 | 147 | 209 | 568.4 | 207 |  |
| 2​ | 85 | 6.1 | 85 | 147 | 271.5 | 147 | 208 | 535.1 | 210 |  |
| 3​ | 85 | 6.1 | 85 | 144 | 242.4 | 154 | 204 | 530.6 | 207 |  |
| 4​ | 87 | 15.6 | 85 | 146 | 275.3 | 145 | 209 | 586.6 | 217 |  |
| 5​ | 86 | 12.5 | 86 | 148 | 302.2 | 145 | 213 | 617.7 | 217 |  |
| **6**​ | **94** | **22.6** | **88** | **151** | **312.5** | **145** | **211** | **569** | **211** |  |
| 7​ | 86 | 12.5 | 88 | 151 | 322.6 | 154 | 211 | 569 | 211 |  |
| 8​ | 86 | 8.3 | 85 | 152 | 282.8 | 158 | 211 | 518.7 | 209 |  |
| 9​ | 85 | 5.5 | 85 | 150 | 276.1 | 154 | 206 | 458.2 | 209 |  |
| **10**​ | **85** | **5.5** | **85** | **154** | **305.3** | **154** | **216** | **570.8** | **217** |  |
| 11​ | 86 | 8.3 | 85 | 148 | 259.9 | 156 | 209 | 516.9 | 216 |  |
| 12​ | 80 | 2.1 | 80 | 145 | 294.8 | 149 | 206 | 588.5 | 206 |  |
| 13​ | 84 | 5.6 | 84 | 142 | 247.2 | 149 | 197 | 624.9 | 206 |  |
| 14​ | 92 | 21.4 | 77 | 149 | 316 | 149 | 213 | 649.5 | 211 |  |
| 15​ | 101 | 33.3 | 82 | 147 | 280 | 149 | 215 | 706.5 | 211 |  |
| 16​ | 84 | 8 | 84 | 141 | 250.6 | 150 | 209 | 711.2 | 206 |  |
| 17​ | 82 | 2.8 | 84 | 143 | 282.6 | 150 | 206 | 630 | 207 |  |
| 18​ | 92 | 24.9 | 88 | 147 | 328.4 | 143 | 210 | 661.9 | 208 |  |
| **19**​ | **100** | **26.9** | **100** | **151** | **224.5** | **153** | **214** | **649.8** | **207** |  |
| 20​ | 104 | 32.7 | 88 | 153 | 291.7 | 153 | 212 | 545.6 | 207 |  |
| 21​ | 99 | 18.3 | 85 | 151 | 260.3 | 155 | 216 | 605.2 | 215 |  |
| 22​ | 102 | 19.4 | 85 | 151 | 259.2 | 155 | 209 | 505.5 | 209 |  |
|  |  |  |  |  |  |  |  |  |  |  |
