# Supplementary material for: A sensorless, Big Data based approach for phenology and meteorological drought forecasting in vineyards
Source: Sci Rep. 2023 Oct 5;13:16818. doi: 10.1038/s41598-023-44019-4 (PMC10556084; doi:10.1038/s41598-023-44019-4)
Supplement: Supplementary file 4 — Supplementary Table S4. [file 41598_2023_44019_MOESM4_ESM.docx]

**Table S4**. Equations and accuracy (R^2^) of multiple-quadratic and linear regression models used for estimating canopy transpiration (T_c_, Y) based on total direct light (TDL, X1), air vapor pressure deficit (VPD, X2) and total canopy light interception (TCLI, X3). Units were: g hour^-1^ for T_c_; µmol m^-2^s^-1^ for TDL; kPa for VPD and unit fraction for TCLI. Calculations refer to the three representative dates of 20 June, 2 July and 1 August.

| **Date** | **Model** | **Equation** | **R^2^ value** | **Variables** |
| --- | --- | --- | --- | --- |
| 20 June | Quadratic-three variables | Y = 7.62563e-09*X1^2+0.0104358*X2^2+1.49096e-06*X3^2+-7.72001e-06*X1*X2+1.07106e-07*X1*X3+-0.000129436*X2*X3 | 0.964 | Y = T_c_; X1 = TDL; X2 = VPD; X3 = TCLI |
| “ | Linear | Y = 0.0712X1 + 146.44 | 0.207 | Y = T_c_  X1 = TDL |
| “ | Linear | Y = 136.57X2 – 147.26 | 0.898 | Y = T_c_  X2 = VPD |
| “ | Linear | Y = -111.74X3 + 321.42 | 0.101 | Y = T_c_  X3 = VPD |
| 2 July | Quadratic-three variables | Y = -2.88612e-08*X1^2+-0.0038447*X2^2+-3.08502e-06*X3^2+4.12417e-05*X1*X2+-7.19364e-07*X1*X3+0.00044962*X2*X3 | 0.939 | Y = T_c_; X1 = TDL; X2 = VPD; X3 = TCLI |
| “ | Linear | Y = 0.0638X1 + 198.36 | 0.203 | Y = T_c_  X1 = TDL |
| “ | Linear | Y = 165.71X2 – 184.4 | 0.887 | Y = T_c_  X2 = VPD |
| “ | Linear | Y = -1.0199X3 + 357.33 | 0.087 | Y = T_c_  X3 = VPD |
| 1 August | Quadratic-three variables | Y = -5.0084e-09*X1^2+0.00716466*X2^2+4.62933e-06*X3^2+-2.40068e-06*X1*X2+1.60601e-07*X1*X3+-0.000160624*X2*X3 | 0.978 | Y = T_c_; X1 = TDL; X2 = VPD; X3 = TCLI |
| “ | Linear | Y = -0.046X1 + 265.15 | 0.180 | Y = T_c_  X1 = TDL |
| “ | Linear | Y = 74.913X2 + 12.871 | 0.554 | Y = T_c_  X2 = VPD |
| “ | Linear | Y = 0.3171X3 + 220.03 | 0.006 | Y = T_c_  X3 = VPD |
